# Macrophage transplantation rescues RNASET2-deficient leukodystrophy by replacing deficient microglia in a zebrafish model

**DOI:** 10.1073/pnas.2321496121

**Published:** 2024-05-16

**Authors:** Holly A. Rutherford, Diogo Candeias, Christopher J. A. Duncan, Stephen A. Renshaw, Noémie Hamilton

**Affiliations:** ^a^Department of Infection and Immunity, School of Medicine and Population Health, University of Sheffield, Sheffield S10 2RX, United Kingdom; ^b^Bateson Centre, University of Sheffield, Sheffield S10 2TN, United Kingdom; ^c^Department of Biology, University of York, York YO10 5DD, United Kingdom; ^d^York Biomedical research Institute, University of York, York YO10 5DD, United Kingdom; ^e^Immunology and Inflammation Theme, Translational and Clinical Research Institute, Newcastle University, Newcastle NE2 4HH, United Kingdom; ^f^Department of Infection and Tropical Medicine, Royal Victoria Infirmary, Newcastle upon Tyne Hospitals National Health Services Foundation Trust, Newcastle NE2 4HH, United Kingdom

**Keywords:** microglia, leukodystrophy, zebrafish, transplantation, white matter disease

## Abstract

RNASET2-deficient leukodystrophy is a rare infantile white matter disorder mimicking a viral infection and resulting in severe psychomotor impairments. Despite its severity, there is little understanding of cellular mechanisms of pathogenesis and no treatments. Recent research using the *rnaset2* mutant zebrafish model has suggested that microglia may be the drivers of the neuropathology, due to their failure to digest apoptotic debris during neurodevelopment. Therefore, we developed a strategy for microglial replacement through transplantation of adult whole kidney marrow–derived macrophages into embryonic hosts. Using live imaging, we revealed that transplant-derived macrophages can engraft within host brains and express microglia-specific markers, suggesting the adoption of a microglial phenotype. Tissue-clearing strategies revealed the persistence of transplanted cells in host brains beyond embryonic stages. We demonstrated that transplanted cells clear apoptotic cells within the brain, as well as rescue overactivation of the antiviral response otherwise seen in mutant larvae. RNA sequencing at the point of peak transplant-derived cell engraftment confirms that transplantation can reduce the brain-wide immune response and particularly, the antiviral response, in rnaset2-deficient brains. Crucially, this reduction in neuroinflammation resulted in behavioral rescue—restoring *rnaset2* mutant motor activity to wild-type (WT) levels in embryonic and juvenile stages. Together, these findings demonstrate the role of microglia as the cellular drivers of neuropathology in *rnaset2* mutants and that macrophage transplantation is a viable strategy for microglial replacement in the zebrafish. Therefore, microglia-targeted interventions may have therapeutic benefits in RNASET2-deficient leukodystrophy.

## Significance

Microglia—the resident immune cells of the central nervous system—are essential to the development and function of the brain. However, microglial dysfunction has been linked to many neurodegenerative and neurodevelopmental disorders, including leukodystrophies—a family of rare but devasting genetic disorders, many of which affect children and have highly limited treatment options available. Here, we demonstrate that transplantation of macrophages can replace deficient microglia in a zebrafish model of leukodystrophy. We show that transplantation can rescue brain-wide neuroinflammation and restore motor function and survival in leukodystrophy-associated mutants. Together, our data identifies a cellular mechanism whereby deficient microglia are the principal drivers of neuropathology in this disorder and supports therapeutic targeting of microglia in the development of treatments for leukodystrophy.

As the resident immune cells of the brain, microglia are essential to the development and function of a healthy nervous system. These highly specialized tissue-resident phagocytes have a host of functions involved in central nervous system (CNS) homeostasis, including phagocytosing debris and dying cells, mediating the immune response through release of carefully balanced pro- and anti-inflammatory mediators, and supporting the health of neurons and other glial cells (1–6). It is perhaps unsurprising, therefore, that microglial dysfunction has been linked to a multitude of disorders, including Alzheimer’s disease, Parkinson’s disease, and white matter diseases (7–11). Although white matter is composed predominantly of myelin—an insulating protein produced and stored within the expansive membranes of oligodendrocytes—it is becoming increasingly apparent that microglial dysfunction can contribute to such diseases, both by directly impacting myelin health and indirectly, by promoting neuroinflammation.

One family of white matter diseases in which the contribution of microglia is becoming more apparent is the leukodystrophies—a family of monogenic white matter disorders, many of which present during infancy (8, 12). These devastating conditions are associated with extensive psychomotor impairment, the formation of white matter lesions, neuroinflammation, and in many cases, limited life expectancy. Although the mechanisms of pathology are, in many instances, specific to the gene affected in each leukodystrophy, microglial dysfunction has begun to emerge as a common theme—even in disorders in which the mutation is thought to affect a gene primarily associated with the function of other glial cell types (8, 13–15). In particular, microglial activation has been suggested to be the first marker—and therefore potential driver—of pathology in animal models of multiple leukodystrophies, which has been corroborated by several postmortem reports from patients (13, 16, 17). In addition, hematopoietic stem cell (HSC) transplantation—which may act to replace microglia—is a clinically established therapy in several leukodystrophies (18–25). As such, microglia-targeted approaches may represent a promising therapeutic avenue in the leukodystrophies.

Here, we show that the addition of healthy microglia reduces both neuropathology and symptom presentation in a zebrafish model of RNASET2-deficient leukodystrophy—a disorder which sits at the intersection of leukodystrophies, lysosomal storage diseases, and a family of autoinflammatory disorders characterized by upregulation of the interferon response, known as interferonopathies (26–28). We have previously demonstrated that a failure of *rnaset2* mutant microglia to clear developmental apoptosis contributes to downstream pathology— leading to an elevated antiviral response, behavioral abnormalities, and reduced survival in the zebrafish model (26). We therefore hypothesize that microglia are the cellular drivers of neuropathology in this disorder and that replacing these deficient cells will rescue *rnaset2* mutant phenotypes. In this study, we develop a methodology to replace deficient microglia by macrophage transplantation and demonstrate that transplantation of macrophages can replenish the microglial population in both healthy and *rnaset2* mutant zebrafish. Moreover, replacing deficient microglia in *rnaset2* mutants is sufficient to rescue clearance of apoptotic debris, antiviral immune responses, and defects in locomotion during embryonic stages. RNA sequencing and behavioral assays confirmed the therapeutic effect of transplanted cells in later stages, with the complete rescue of neuroinflammation and impaired swimming. As such, our findings provide further evidence that microglia are central to RNASET2-deficient leukodystrophy pathology and support a targeting of microglia as a potential therapeutic strategy.

## Materials and Methods

### Animal Models.

All procedures involving zebrafish followed the Animal (Scientific Procedures) Act 1986 under the Home Office Project Licence (PPL P254848FD). Adult animals were raised and maintained in the Biological Services Aquarium under careful monitoring at 28 °C under a 14/10 h light/dark regimen. Zebrafish embryos were maintained in Petri dishes of approximately 60 embryos in E3 medium in light cycling incubators at 28 °C until 5 days postfertilization (dpf).

*rnaset2^sh532^* mutant fish were previously generated using CRISPR/Cas9 genome editing technology in *Tg(mpeg1:mCherryCAAX)sh378* embryos with founders identified and bred to produce a stable line (26, 29). Wild-type (WT) and homozygous adults were identified using fin clip genotyping and isolated for breeding. *csf1ra^−/−^;csf1rb^+/−^* mutants were incrossed to generate double-mutant offspring and were genotyped as previously described (30, 31). For isolation of GFP-positive macrophages, *Tg(fms:GFP)sh377* animals were used (32).

### Zebrafish-to-zebrafish Immune Cell Transplantation.

To prepare grafts for transplantation, whole kidney marrow was isolated from ten transgenic adult fish with GFP-labeled macrophages *Tg(fms:GFP)sh377* and GFP-negative controls (nacre). Each kidney was then placed in 200 μL of cold live sorting buffer (L15 medium, 20% foetal bovine serum and 5 mM EDTA) and mechanically separated using repeated pipetting. Samples from each fish were kept separate, and on ice, until immediately before flow cytometry to prevent cross-reactivity or immune activation. Using a BD FACSMelody^TM^ cell sorter (BD Biosciences) at 4 °C, GFP-negative samples were sorted first to determine thresholds for GFP-fluorescence, and TO-PRO^TM^3 (ThermoFisher, R37170) was used to facilitate removal of dead cells. GFP-positive cells were sorted into an Eppendorf tube containing 500 μL live sorting buffer and placed on ice before transplantation. Cell yields of approximately >500,000 cells were optimum for transplantation. Following fluorescence-activated cell sorting (FACS), we noted that sorted cells were transiently photobleached, with reduced GFP visibility at the time of transplantation which returned following engraftment. Therefore, to allow immediate visualization, sorted cells were additionally labeled with the fluorescent dye carboxyfluorescein succinimidyl ester (CFSE) by incubation in 1:10,000 CFSE dilution for 15 min at 26 °C. Cells were washed and suspended in 1% polyvinylpyrrolidone (PVP) in live sorting buffer, before final centrifugation and resuspending at a concentration of approximately 20 to 30 cells per nanoliter.

Transplants were performed on 2 dpf *Tg(mpeg1:mCherry CAAX)sh378* embryos, which have endogenous microglia and macrophages labeled with mCherry, or *csf1ra^−/−^;csf1rb^+/−^* incrossed embryos. These embryos had either undergone microglia depletion via CRISPR/Cas9-mediated targeting of *irf8*, or injection of scrambled control guide RNA (described below). On the day of transplantation, hosts were dechorionated and sorted cell grafts were injected into the systemic circulation via the duct of Cuvier following sedation with 4.2% tricaine. Hosts were then recovered from anesthesia in fresh E3 and returned to the incubator for recovery. As a negative control, approximately 60 embryos received a sham transplant containing only 1% PVP in live cell sorting buffer, without any cells present.

Following transplantation, embryos were sedated with 4.2% tricaine and screened using a Zeiss Axioscope to confirm the presence of GFP-positive cells. For longitudinal quantification of transplanted cell count within each host, positive embryos were screened at 3 dpf and transferred to individual wells of 48-well plates for follow-up. For all other assays, embryos were screened at 5 dpf and those with 10 or more GFP-positive cells in their brain were taken forward for subsequent assays. For raising until 8 dpf, embryos were transferred to fresh Petri dishes containing E3 medium and returned to the light cycling incubator. Medium was changed daily, along with the addition of fresh food and removal of any unhealthy larvae for culling by schedule 1. For raising to adulthood, embryos were housed at densities of 15 to 20 animals per tank and fed twice daily to ensure healthy development.

### Generation of irf8 Crispants to Deplete Embryonic Microglia.

To deplete embryonic microglia from hosts at the point of transplantation, CRISPR/Cas9 genome editing was utilized to target interferon regulatory factor-8 (*irf8*)—a transcription factor essential for the development of embryonic macrophages and microglia through primitive and transient definitive hematopoiesis but not adult-phase definitive hematopoiesis (33, 34). To achieve maximum depletion, two guides targeting *irf8* expression (*SI Appendix*, Table S1) were injected at a final concentration of 50 μM each by creating an injection mix of 0.5 μL of each guide at 100 μM, 1 μL of 50 μM tracer, and 1 μL Cas9 nuclease (New England Biolabs, MO386M). As a control, scrambled crRNA was used at 50 μM in the place of *irf8*-targeting guides. For each embryo, 2 μL of injection mix was injected into the yolk sac at the single cell stage. Fish were screened for survival using light microscopy each day following injection, with unhealthy or dead embryos removed. To assess microglia depletion, the number of *mpeg:mCherry* cells in the brains of *irf8* crispants were quantified at 5 dpf using ZEISS Axio Scope (*SI Appendix*, Fig. S1 *A* and *B*). For transplantation experiments, embryos with less than 10 *mpeg1*:*mCherry* cells in the brain at 5 dpf were selected.

### Whole Mount TUNEL and Immunohistochemistry.

In order to assess the functionality of transplanted cells in *rnaset2* mutant embryos at 5 dpf and 8 dpf, TUNEL staining (to visualize uncleared apoptotic debris) and immunohistochemistry (to visualize expression of microglia-specific markers) were performed in parallel on the same samples.

Following screening to ensure transplanted cell engraftment and effective microglia depletion (if employed), 5 dpf and 8 dpf embryos were immersion fixed in 4% paraformaldehyde overnight at 4 °C following terminal sedation in concentrated tricaine. Following fixing, samples were rinsed with PBST and dehydrated with increasing concentrations of methanol (25% MeOH:75% PBS; 50% MeOH:50% PBS; 75% MeOH:25% PBS; 100% MeOH) and stored at –20 °C until TUNEL assay and/or immunohistochemistry.

To quantify apoptosis, we utilized the terminal deoxynucleotidyl transferase (TdT)-mediated dUTP nick end labeling (TUNEL) assay (ApopTag® Red In Situ Apoptosis Detection Kit, Sigma-Aldrich, S7165), as per manufacturer instructions. Directly after the completion of the TUNEL, embryos were incubated in primary antibody solution for 48 h at 4 °C at following concentrations: anti-4C4 mouse antibodies at 1:100 (35) and anti-GFP chicken antibodies (GeneTex, GTX13970) at 1:500. Anti-4C4 primary was isolated from 7.4.C4 mouse hybridoma cells (Sigma, 92092321-1VL) as per manufacturer instructions. Samples were then washed and incubated in the relevant secondary antibodies for 24 h at 4 °C: Alexa Fluor^TM^ 647 goat anti-mouse (Thermo-Fisher Scientific, A-21235) and Alexa Fluor^TM^ 488 goat anti-chicken (Thermo-Fisher Scientific, A-11039).

**Fig. 1.**
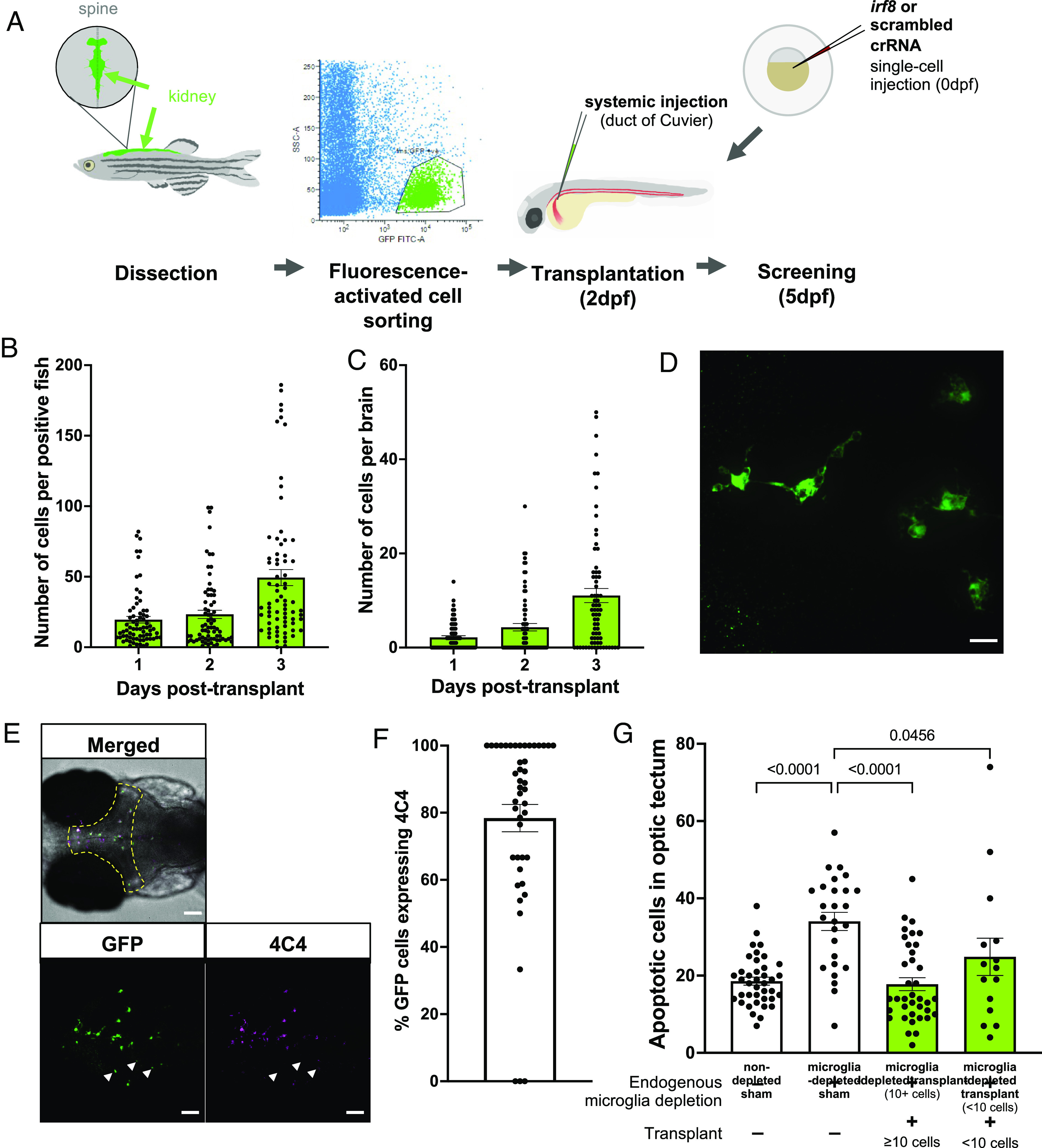
Macrophage transplantation successfully replaces microglia in WT zebrafish. (*A*) Transplants were performed by dissecting kidneys from *Tg(fms:GFP)* adult donors and isolating GFP-positive cells using fluorescence-activated cell sorting. Cells were injected into 2 dpf *Tg(mpeg1:mCherryCAAX)sh378* hosts via duct of Cuvier. Hosts had previously undergone microglia depletion via targeting of *irf8* by CRISPR/Cas9 or were nondepleted with a scrambled control. (*B* and *C*) The number of *fms:GFP* cells per successfully transplanted animal increased throughout the whole body (*B*) and within the brain (*C*) across 3 d post-transplant, as revealed by manual counting. (*D*) Confocal imaging reveals that transplant-derived cells show a branched, microglia-like morphology. The scale bar represents 17 µm. (*E* and *F*) Transplant-derived cells express the microglia-specific marker 4C4 at 3 d post-transplant (5 dpf). Colocalization was assessed by manual counting. Arrows indicate GFP-positive cells that do not co-localize with 4C4. The yellow dashed line indicates the border of the optic tectum (region of interest). Three biological replicates, n = 45. The scale bar represents 75 µm. (*G*) TUNEL staining reveals that transplantation can rescue the number of uncleared apoptotic cells in microglia-depleted brains in a dose-dependent manner, as revealed by manual counting of cells within optic tectum as in *E*. Kruskal–Wallis test with Dunn’s multiple comparisons. Four biological replicates, n = 15 to 39.

Immunostained embryos were mounted in low melting temperature agarose and imaged using Nikon W1 Spinning Disk. Each embryo was imaged using 20× magnification, with a Z-stack of 50 slices (2 µm per slice). Maximum intensity projections generated using Fiji are shown throughout. Imaging and quantification were performed blinded to minimize bias. To ensure consistent counting, the optic tectum was selected as the region of interest and was identified using a brightfield reference image for each sample (Fig. 1*E*). TUNEL counts and 4C4-GFP colocalization were then counted manually using Fiji.

### Tissue Clearing.

Tissue clearing and immunohistochemistry methodology was adapted from Susaki et al. (36) and Ferrero et al.(37). Imaging of microglia in cleared brains was optimized using *Tg(fms:GFP)sh377* and *Tg(mpeg1:mCherryCAAX)sh378* animals (*SI Appendix*, Fig. S2). Detailed protocol and semiautomated image analysis pipeline can be found in *SI Appendix*, *Method* section.

### Larval RNA Extraction and cDNA Synthesis.

Prior to RNA extraction, embryos (5 to 8 dpf) were culled by terminal anaesthesia in concentrated tricaine, before being transferred to Eppendorf tubes and the addition of 500 µL Trizol reagent (Invitrogen, 15596026). Once in the Trizol reagent, culled embryos were homogenized using a handheld homogenizer (T 10 basic ULTRA-TURRAX, IKA Dispersers, product no. 0003737002) and RNA extracted using the chloroform/isopropanol method and the resultant pellet was dissolved in 20 µL nuclease-free water to be used for cDNA synthesis. cDNA was synthesized utilizing Superscript II according to the manufacturer’s instructions using 2 µg of RNA input, and the resultant cDNA was diluted 1:20 for qPCR.

### qRT-PCR.

qRT-PCR was performed to assess relative gene expression using the primers listed in *SI Appendix*, Table S2. Reaction mixtures were as follows: 5 μL SYBR green, 2 μL milli Q water, 2 μL cDNA, 0.5 μL forward primer, and 0.5 μL reverse primer (both 10 µM). The qRT-PCR was run on a CFX96 Bio-Rad machine as follows: step 1: 95 °C for 2 min, step 2: 95 °C for 10 s, step 3: 57 °C for 30 s, step 4: 72 °C for 25 s, step 5: 95 °C for 30 s, step 6: 65 °C for 10 s, step 7: 95 °C for 20 s with steps 2 to 4 repeated 39 times and increment of 0.2 °C every 10 s between steps 6 and 7. As previously published, ef1α was used as a reference gene for each sample, each reaction was performed in triplicate, with the mean Cq value for each reaction used to determine relative expression (26).

### Larval Free-swimming Analysis.

In order to assess larval free-swimming behavior, embryos were raised to 8dpf under approved individual study plans. Briefly, animals were raised in Petri dishes—12 embryos per dish—containing E3 medium which was refreshed daily by the personal license holder. Embryos were fed daily, using a Pasteur pipette to ensure uniform distribution of food between plates. On the morning of the experiment, embryos were transferred to 24-well plates (one embryo per well) without anaesthetizing, with 500 µL clear E3 per well. Plate layout was alternated between experimental groups to ensure no location-specific effects—and allowed to habituate for at least 4 h. Swimming distance was recorded across a 20 to 60-min period, with alternating light–dark cycles of 10 min. Outliers were manually excluded based upon movement analysis trace automatically generated by Zebralab software.

### Juvenile Free-swimming Analysis.

For juvenile swimming behavior analysis, fish were raised as normal to 4 wk postfertilization (wpf). Animals were transferred to the behavioral facility on the morning of recording and allowed an initial 30-min habituation period to recover from any stress when transferring between rooms. For recording, each adult was placed in an individual 0.7L tank (Techniplast) containing 200 mL aquarium water. The walls of each tank were covered with white paper to ensure that the animals were unable to interact or be distracted by their surroundings.

After transferring to the behavioral tanks, fish were given a further 10 min to habituate to their environment, before recording for a period of 10 min. Recording was performed using the Basler GenICam camera, and tracking was performed using EthoVision XT15 (advanced detection settings with dynamic subtraction). The total distance swum was extracted from EthoVision software.

Following recording, animals were either culled by schedule 1 or by decapitation (regulated K) if their brains were required for further experiments. Animals were size-matched throughout. At the 4 wpf timepoint, animal sex could not be determined due to their small size and early development.

### Survival Analysis.

Survival at 4 wpf was defined as the absence of any observable sickness behaviors. Animals were routinely monitored, and any animal with the inability to swim to feed was humanely culled.

### RNA Sequencing.

For RNA sequencing, 4 wpf transplanted *rnaset2* mutants and sham controls were culled and brains were dissected as previously described. Dissected brains were placed immediately into liquid nitrogen to snap-freeze the tissue and preserve RNA integrity for extraction at a later timepoint. RNA was then extracted using Trizol as described above, except for the final step in which the resulting RNA pellet was dissolved in 100 µL nuclease-free water (rather than 10 µL) to aid column purification.

The RNeasy MinElute Cleanup Kit (Qiagen, cat. no. 74204) was used to purify RNA for sequencing following the manufacturer’s instructions and resuspended in 14 µL nuclease-free water before being stored at –80 °C. Quality control was performed by The Genomics Laboratory at the University of York, using the Agilent BioAnalyzer 2100. Samples with an RNA integrity number (RIN) above 7.0 were taken forward for sequencing.

Library preparation was performed by The Genomics Laboratory at the University of York, using the NEBNext Ultra II Directional Library prep kit for Illumina in conjunction with the NEBNext® Poly(A) mRNA Magnetic Isolation Module and unique dual indices (New England Biolabs), according to the manufacturer’s instructions. Libraries were pooled at equimolar ratios and sent for paired-end 150 base sequencing at Azenta Life Sciences on an Illumina NovaSeq 6000 instrument.

Reads (data accession number PRJNA1047321) were trimmed using Cutadapt v3.4 (38) and then mapped to the GRCz11 genome using Spliced Transcripts Alignment to a Reference v2.7.10b (39). Counts were generated for each gene using htseq-count v2.0 (40). Differential expression analysis was performed using DESeq2 (41) using three-way comparisons between WT sham, *rnaset2* sham, and *rnaset2* transplanted samples (with separate analyses for microglia-depleted and nondepleted groups). Pathway analysis was performed using g: Profiler (https://biit.cs.ut.ee/gprofiler/gost) (42). For GSEA, genes were ranked according to their Wald statistic results from the differential expression analysis. The ranked list of genes was then used for a GSEA using the package *fgsea* v1.28.0 and the results were plotted using the package *ggplot2* v3.4.4. in R v4.3.2.

### Statistical Analysis.

All statistical analysis was performed using GraphPad Prism, with the exception of RNA sequencing. For pairwise comparisons, data were entered using a column table (two samples, one variable only) and analyzed using Mann–Whitney test. For comparisons between more than two samples, data were entered using a grouped table and analyzed using Kruskal–Wallis test with Dunn’s multiple comparisons. For survival analysis, log-rank Mantel–Cox test with Bonferroni’s multiple comparison correction was used. Exact *P* values are stated throughout.

For larval experiments, assays were repeated three times using different batches of larvae born on different dates, with the number of biological replicates and n (experimental unit) number stated for each experiment in figure legends unless otherwise stated. For juvenile experiments, each animal represents an experimental unit (n stated in figure legend).

For RNA sequencing, significance testing was performed using Wald tests with Benjamini–Hochberg p value correction, as previously described (41).

## Results

### Macrophage Transplantation Successfully Replaces Microglia in WT Zebrafish.

We have previously demonstrated that *rnaset2* microglia are deficient in their ability to clear apoptotic debris—leading to a downstream neuroinflammatory response (26). As such, we sought to replace these dysfunctional microglia using transplantation of healthy macrophages. Although HSC transplantation is used clinically in hereditary leukodystrophies and may act to replace microglia, transplanted macrophages may represent a quicker route to repopulation of the brain—minimizing the need for HSC engraftment and instead migrating directly to the CNS. To this end, healthy macrophages were isolated from whole kidney marrow from *Tg(fms:GFP)* adult fish and purified by FACS. Purified macrophages were injected at 2 d postfertilization (dpf) into embryos with endogenous embryonic macrophages depleted by CRISPR/Cas9 injection of guide RNA targeting the transcription factor *irf8*, which is essential for the development of macrophages by primitive and transient definitive hematopoiesis (Fig. 1*A*) (34). This approach allowed the depletion of microglia at the time of transplantation in crispant hosts (*SI Appendix*, Fig. S1). Transplanted macrophages were able to robustly engraft within host embryos and reach the brain within three days of transplant in microglia-depleted animals (Fig. 1 *B* and *C*). The number of transplanted cells in the brain increased in the days post-transplant, with timelapse imaging confirming that transplanted macrophages were able to divide following engraftment in the brain (Fig. 1*C* and Movie S1). Macrophage transplantation was well tolerated, with little mortality in transplanted hosts up to 5 dpf (*SI Appendix*, Fig. S3). Conversely, transplantation of CD41-positive hematopoietic stem cells was less efficient thereby confirming the suitability of whole kidney marrow-derived macrophages as a graft resulting in a more direct repopulation of the microglial niche (*SI Appendix*, Fig. S4). Depleting the host of endogenous microglia using *irf8* crRNA CRISPR/Cas9 injection promoted transplanted cell engraftment, highlighting the importance of emptying the niche before transplantation (*SI Appendix*, Fig. S4 *A* and *B*)**.**

Transplanted macrophages displayed a microglia-like morphology with multiple branches (Fig. 1*D*). As such, we sought to investigate whether these cells could adopt aspects of the microglial phenotype following engraftment. Using colocalization with the microglia marker 4C4 (35), we showed that up to 80% of transplant-derived cells express microglia-specific proteins—suggesting that these whole kidney marrow–derived macrophages are able to adapt to their host environment (Fig. 1 *E* and *F*). Crucially, transplantation was associated with reduced abundance of apoptotic cells in a dose-dependent manner, demonstrating the ability of transplant-derived cells to phagocytose dying cells within the CNS (Fig. 1*G*). Together, these data suggest that transplanted cells are able to reprogramme as microglia and undertake tissue-resident functions.

### Macrophage Transplantation Reduces Early Neuropathology in *rnaset2* Mutants.

**Fig. 2.**
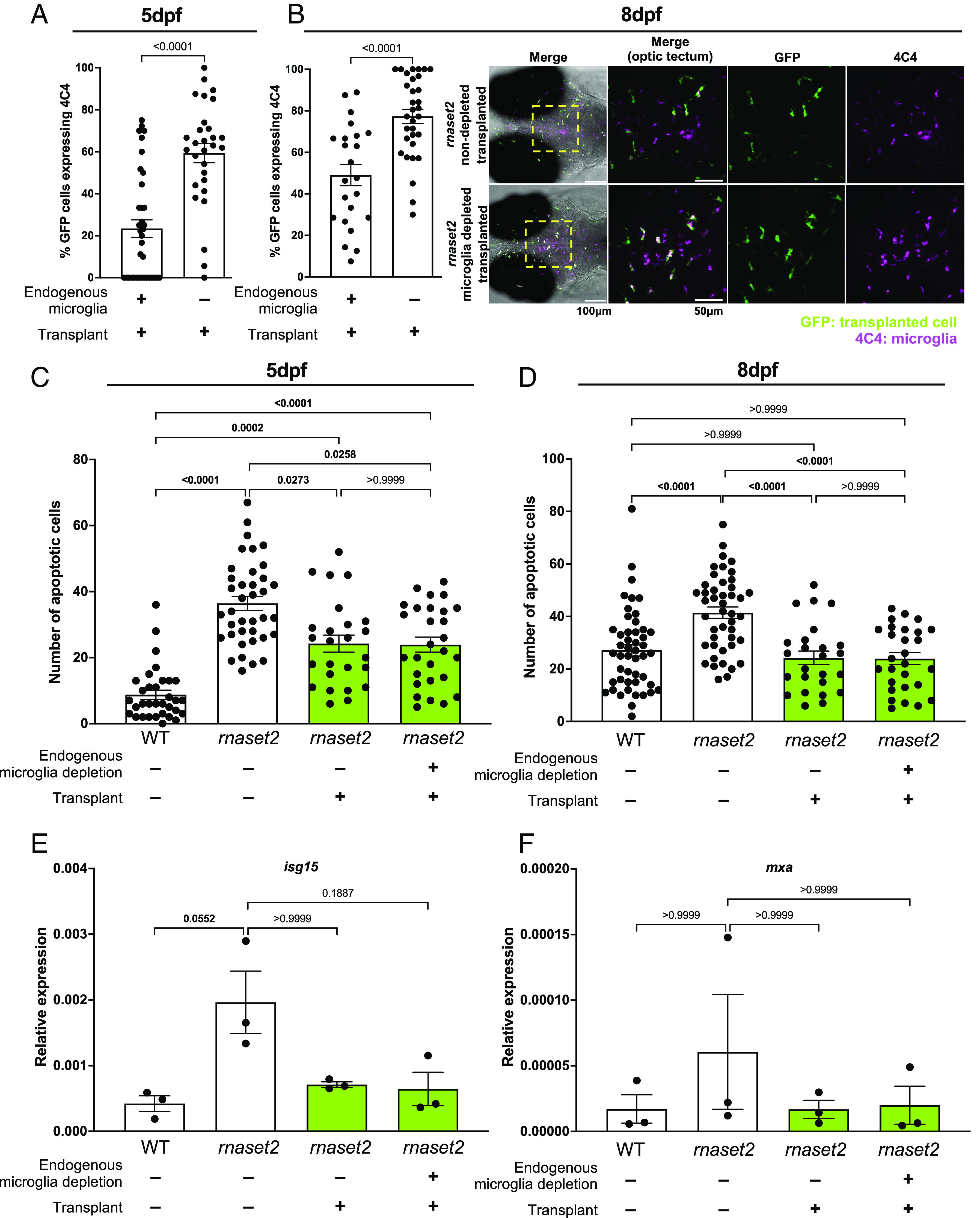
Macrophage transplantation reduces early neuropathology in *rnaset2* mutants. (*A* and *B*) Quantification of 4C4-expression of transplant-derived cells in microglia-depleted versus nondepleted *rnaset2* hosts at 5 (*A*) and 8 dpf (*B*) reveals reduced expression of 4C4 by transplanted cells in nondepleted hosts. Mann Whitney test. Three biological replicates, n = 29 to 37 (*A*), three biological replicates, n = 23 to 32 (*B*). (*C* and *D*) Macrophage transplantation reduces the number of uncleared apoptotic cells in the optic tectum of 5 (*C*) and 8 dpf (*D*) *rnaset2* mutants. Kruskal–Wallis test with Dunn’s multiple comparisons. Four biological replicates, n = 25 to 38 (*C*), three biological replicates, n = 25 to 48 (*D*). (*E* and *F*) qPCR reveals that *isg15* (*E*) and *mxa* (*F*) expression is reduced in transplanted animals, relative to WT controls. Kruskal–Wallis test with Dunn’s multiple comparisons. Three biological replicates, 15 embryos pooled per replicate.

After demonstrating that transplanted macrophages can repopulate host brains in microglia-depleted WT animals, we next sought to investigate whether transplantation had comparable efficacy in *rnaset2* mutants. As *rnaset2* microglia are deficient in their ability to clear apoptotic cells accumulating during development—known to be one of the key drivers of microglial infiltration into the developing brain—we investigated whether depletion of embryonic microglia at the point of transplantation was necessary for complete engraftment (43). Interestingly, we found significantly more transplant-derived cells in the brains of microglia-depleted hosts compared to nondepleted controls, suggesting that transplanted macrophages compete with dysfunctional endogenous microglia cells for brain engraftment, as found in WT brains (*SI Appendix*, Figs. S4 and S5). However, we also noticed that a greater proportion of transplanted cells expressed the microglia-specific marker 4C4 in microglia-depleted animals compared with nondepleted controls—suggesting that these cells are able to take on a microglial-like phenotype in *rnaset2* mutants when endogenous microglia are absent but retain aspects of their macrophage identity when host microglia are present (Fig. 2 *A* and *B*). This failure to express microglia-specific markers was found in transplanted cells in nondepleted brains both 3- and 5-d after transplant—suggesting that this is not simply due to a delay in cells entering the brain where they may undergo reprogramming but rather due to an overall failure to adopt a microglia-like phenotype.

After demonstrating that transplanted macrophages are able to engraft in *rnaset2* embryos, we next sought to investigate whether this intervention could rescue neuropathology in our disease model. While microglia depletion has been shown to increase the number of uncleared apoptotic cells in WT brains, we have previously demonstrated that the apoptotic cell count in *rnaset2* mutants is unchanged with microglia depletion—suggesting that a failure of microglia to digest dying cells during development is one of the earliest factors in *rnaset2* pathology (26). Therefore, we sought to investigate the ability of transplanted macrophages to clear apoptotic debris in *rnaset2* mutants, with and without microglia depletion. Indeed, TUNEL staining revealed that transplanted animals exhibited significantly fewer uncleared apoptotic cells at 5 and 8 dpf, with a complete rescue to WT levels at 8 dpf (Fig. 2 *C* and *D* and *SI Appendix*, Fig. S6). Interestingly, this clearance of apoptotic cells was comparable across transplanted animals regardless of endogenous microglia depletion at both ages. We had previously hypothesized that this bottleneck of apoptotic cell digestion may trigger downstream neuropathology—in particular, the antiviral response which is prominently up-regulated in mutants during larval and adult stages. As such, we next investigated the impact of transplantation on antiviral responses in the heads of 8 dpf animals. qPCR revealed an approximately fourfold downregulation of the interferon response gene *isg15* in transplanted mutants—a correlate of the antiviral response significantly up-regulated in the *rnaset2* mutants (Fig. 2*E*). A similar pattern of rescue was observed for other antiviral genes, including *mxa* (Fig. 2*F*). This normalization of the antiviral response could not be explained by elevated levels of functional, transplant-derived rnaset2, as *rnaset2* transcript levels remained down-regulated in transplanted mutants compared to WT controls (*SI Appendix*, Fig. S7). Therefore, these findings suggest that the presence of phagocytosis-competent macrophages in the CNS can stabilize the antiviral cascade in *rnaset2* brains.

### Long-lasting Transplanted Macrophages Rescue Neuroinflammation beyond Embryonic Stages.

**Fig. 3.**
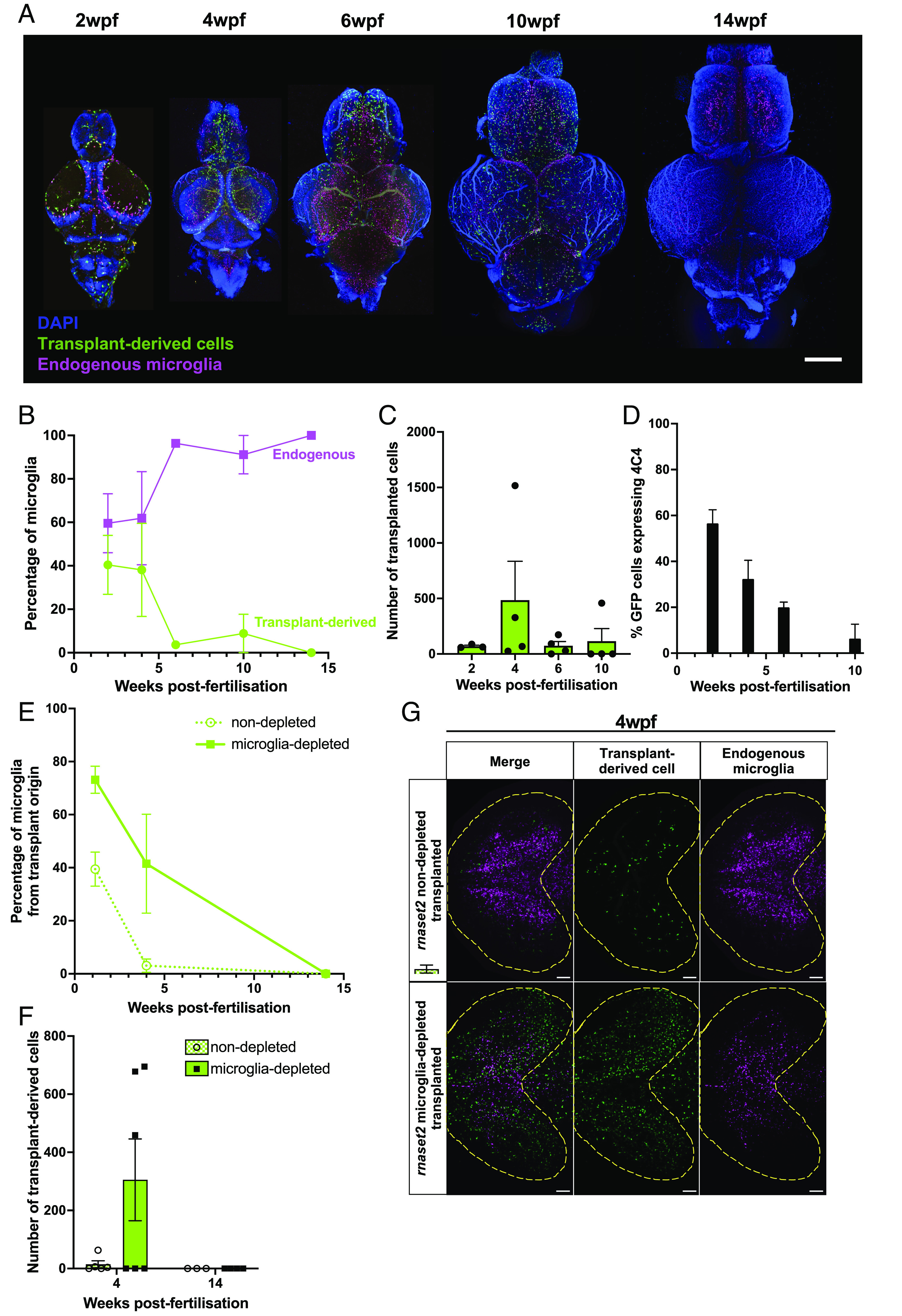
Transplanted cells persist in host brains throughout juvenile stages. (*A*) Tissue clearing and immunohistochemistry reveal that transplant-derived cells persist in WT microglia-depleted host brains until 10 wpf but are cleared by 14 wpf. Scale bar represents 400 µm. (*B*) Percentage of microglia from transplant origin gradually decreases in host brains until 14 wpf. (*C*) The number of transplant-derived cells peaks at 4 wpf and rapidly decreases thereafter. (*D*) Percentage of transplant-derived cells expressing microglia marker 4C4 steadily decreases from 2 wpf. Three to four biological replicates per timepoint. (*E* and *F*) Tissue clearing and immunohistochemistry reveal reduced numbers of transplant-derived cells in nondepleted *rnaset2* animals compared with microglia-depleted siblings. Cells are no longer visible in both host groups by 14 wpf. (*G*) Representative image of transplant-derived cell engraftment at 4 wpf in nondepleted and microglia-depleted *rnaset2* hosts. Yellow line indicates optic tectum outline. The scale bar represents 100 µm.

To assess whether transplanted cells persisted in the brain to provide long-lasting therapeutic effects, we used tissue clearing followed by immunohistochemistry on brains from WT transplanted animals to quantify endogenous versus transplanted cell number over time. We found that transplant-derived cells initially expand in number—peaking at 4 wpf—but were no longer detectable by 14 wpf (Fig. 3 *A*–*C*). This reduction in transplanted cell number appeared to be accompanied by a gradual reduction in the percentage of remaining transplant-derived cells which express the microglial-specific marker 4C4 (Fig. 3*D*). Interestingly, like in microglia-depleted WT hosts, we saw a robust engraftment of transplanted cells maintained in microglia-depleted *rnaset2* brains until 4 wpf, which was also cleared by 14 wpf (Fig. 3 *E*–*G*). However, in nondepleted *rnaset2* mutants, transplanted cells were largely absent by 4 wpf–suggesting that these cells are cleared more quickly when host microglia are present. Together, these data suggest that transplant-derived cells are able to maintain engraftment for the first few months of life in hosts lacking endogenous embryonic microglia, regardless of *rnaset2* genotype.

Embryonic microglia depletion via mutation of *irf8* has been shown to ablate embryonic but not adult microglia (34). Indeed, as anticipated, the number of endogenous microglia at 4 wpf was unchanged in animals who had undergone embryonic microglia-depletion relative to nondepleted controls (*SI Appendix*, Fig. S1 *E* and *F*). In order to assess whether the arrival of adult, definitive hematopoiesis-derived microglia may drive the clearance of transplant-derived cells in the brain, we utilized *csf1ra^−/−^*; *csf1rb^−/−^* (hereafter referent to as *csf1r^DM^*) hosts, which lack endogenous microglia throughout life (31). We found that transplant-derived cells persist up to 26 wpf in *csfr1^DM^*, beyond the point of clearance in *irf8* crispants (*SI Appendix*, Fig. S8). Interestingly, transplant-derived cells were cleared from *csf1ra^−/−^; csf1rb^+/+^* hosts— which lack embryonic, but not adult, microglia—along a similar time course to *irf8* crispants. Therefore, this suggests transplanted cells can persist for longer durations in hosts which completely lack microglia throughout life but may be cleared by endogenous adult microglia when only embryonic microglia depletion is employed. As such, in order to investigate the impact of transplantation on *rnaset2* pathology beyond embryonic stages, we utilized microglia-depleted 4 wpf hosts—the peak of transplanted cell number in our leukodystrophy model—for our subsequent experiments.

**Fig. 4.**
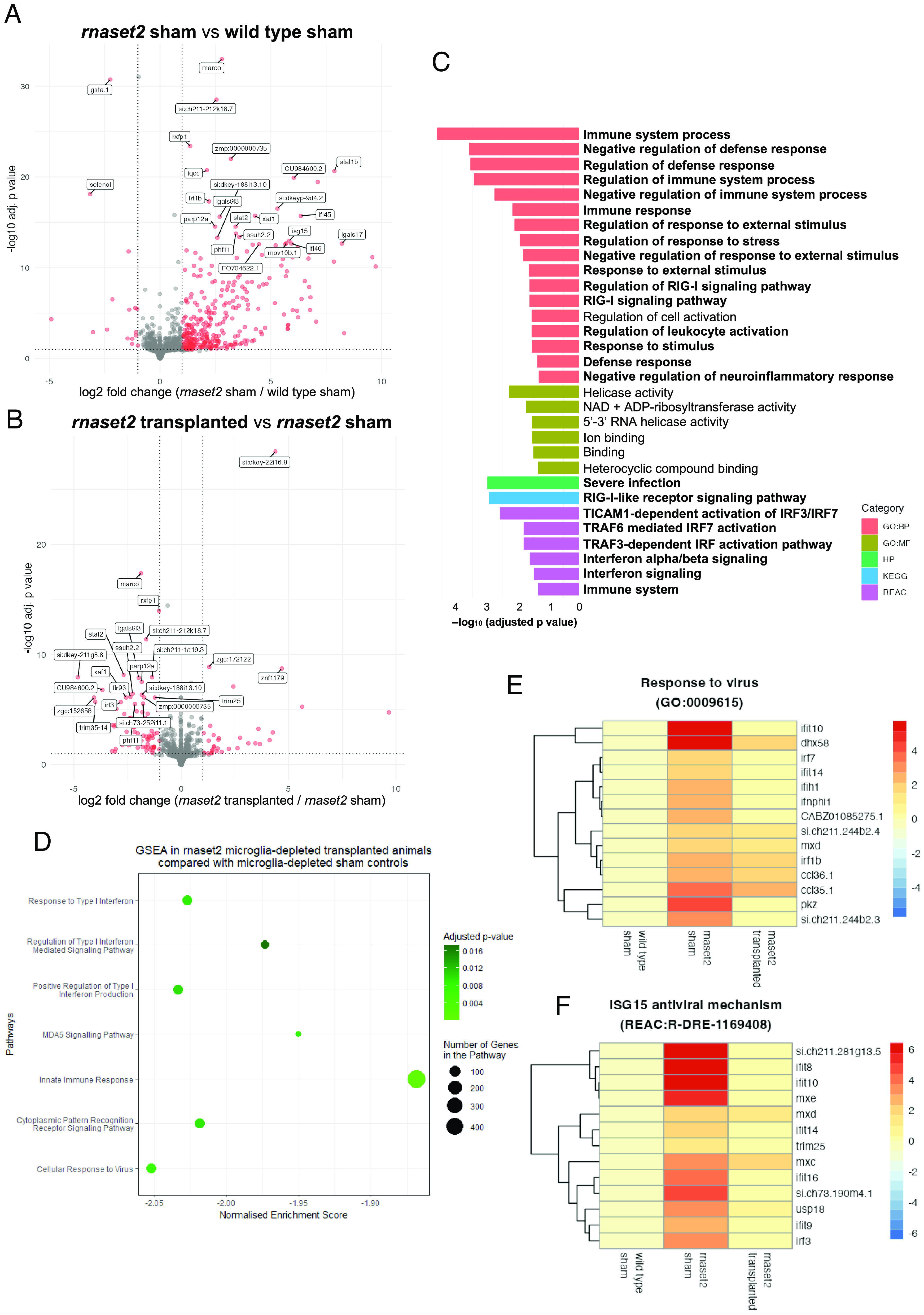
RNA sequencing reveals that microglia replacement rescues antiviral immune response in 4 wpf *rnaset2* mutants. (*A* and *B*) Volcano plot of differentially expressed genes between *rnaset2* sham versus WT sham (*A*) and *rnaset2* transplanted versus *rnaset2* sham groups (*B*). Significantly differentially regulated genes are shown in red. The top 25 differentially expressed genes are annotated. (*C*) Gene ontology (GO) plot showing significantly downregulated pathways in *rnaset2* microglia-depleted transplanted animals compared with microglia-depleted sham controls, identified by gProfiler. Pathways relating to immune and antiviral response are indicated in bold. (*D*) Gene set enrichment analysis (GSEA) in *rnaset2* microglia-depleted transplanted animals compared with *rnaset2* microglia-depleted sham controls. (*E* and *F*) Heatmap of genes belonging to response to virus (*E*) and ISG15 antiviral mechanism (*F*) GO pathways for all genes significantly up-regulated in *rnaset2* sham animals. Fold change relative to WT sham is indicated by color, with red indicating higher expression. All data shown correspond to microglia-depleted animals. See also *SI Appendix*, Fig. S4.

We performed bulk RNA sequencing on brains from *rnaset2* microglia-depleted transplanted animals compared with microglia-depleted sham controls (n = 4 brains per group) (Fig. 4 *A*–*C*). We identified significantly enriched pathways in *rnaset2* microglia-depleted transplanted animals compared with *rnaset2* microglia-depleted sham control (Fig. 4*C*), with a significant rescue in antiviral immune response pathways by gene set enrichment analysis (GSEA) (Fig. 4*D*). Antiviral immune pathways, such as “Response to virus” and “ISG15 antiviral mechanisms,” were restored to WT in transplanted animals (Fig. 4 *E* and *F* and *SI Appendix*, Fig. S9). Interestingly, immune-related pathways were not enriched in *rnaset2* nondepleted transplanted animals compared with nondepleted sham controls, likely due to a lack of transplant-derived cells in host brains at this timepoint (n = 3 brains per group) (*SI Appendix*, Figs. S10 and S11). Nonetheless, this suggests that adding phagocytosis-competent healthy macrophages at early stages in brain development can rescue the brain-wide immune response, specifically suppressing the antiviral immune response, in *rnaset2* mutants.

### Transplantation Rescues *rnaset2* Motor Impairment and Survival beyond Embryonic Stages.

**Fig. 5.**
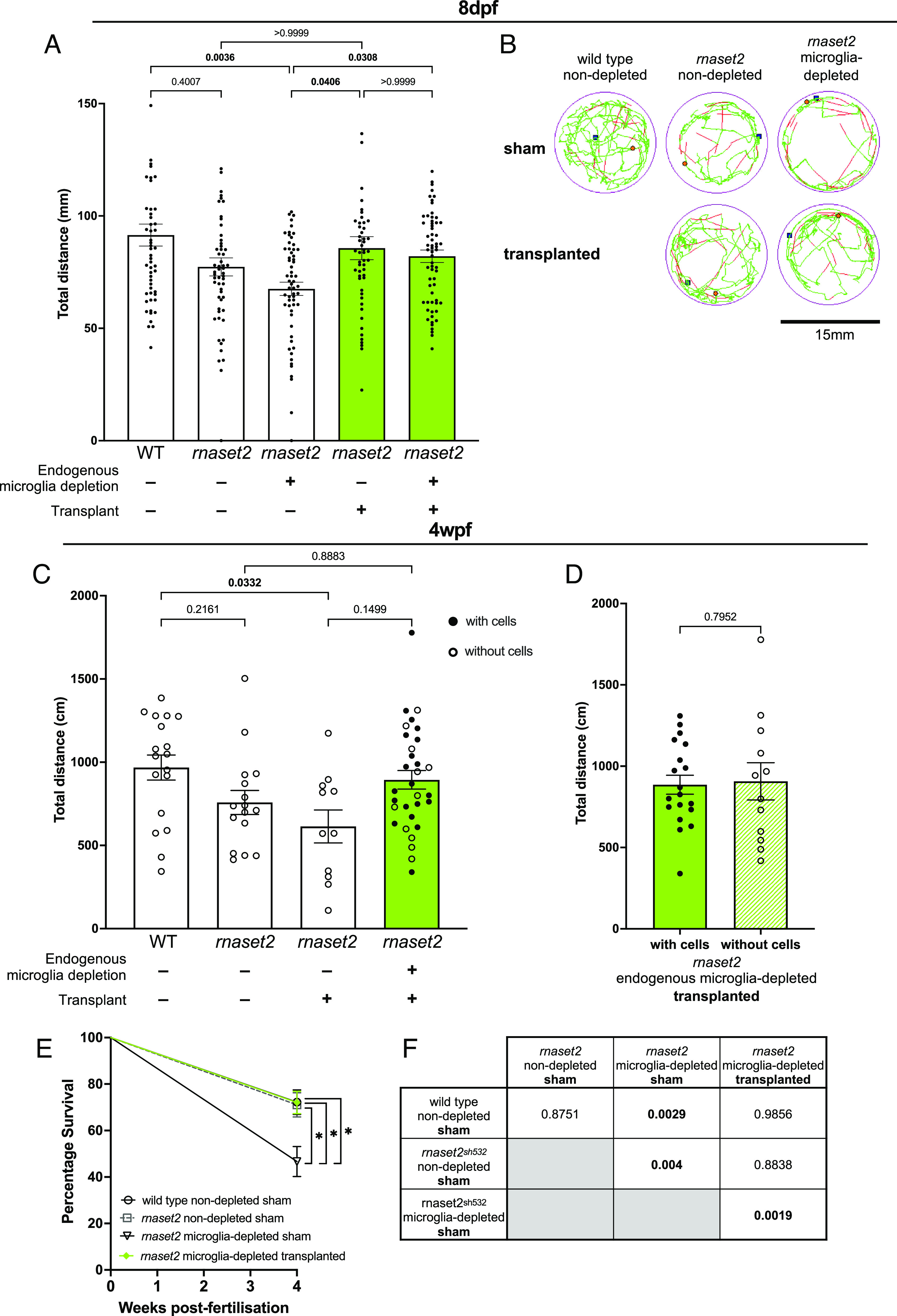
Transplantation rescues *rnaset2* mutant swimming behavior and survival. (*A*) Live tracking of larval swimming behavior reveals that transplanted *rnaset2* mutants swim greater distances than nontransplanted controls. Kruskal–Wallis test with Dunn’s multiple comparisons. Three biological replicates, n = 52 to 60. (*B*) Representative traces of free-swimming behavior of zebrafish larvae. The green line represents slow movements (3.0 to 6.6 mm/s), red line represents fast movements (>6.6 mm/s). (*C*) Tracking of 4 wpf juvenile behavior reveals that transplantation restores *rnaset2* swimming behavior to WT levels. Kruskal–Wallis test with Dunn’s multiple comparisons, n = 11 to 31. (*D*) Swimming behavior was comparable between *rnaset2* microglia-depleted transplanted animals that had persistent engraftment of transplanted cells and those which did not. Mann–Whitney *U* test, n = 12 to 19. (*E* and *F*) Percentage survival of transplanted *rnaset2* mutants is greater than microglia-depleted sham controls at 4 wpf. Pairwise comparisons and corresponding *P* values shown in *F*, bold indicates significance. Log rank Mantel–Cox test with Bonferroni’s multiple comparison correction (Bonferroni-corrected threshold: *P* < 0.00833; family-wise significance threshold: *P* < 0.05). Three biological replicates, n = 60 to 86.

After demonstrating that transplantation rescued many of the hallmarks of *rnaset2* neuropathology, we next sought to investigate whether these changes translated to altered disease-associated behavior. Larval swimming analysis revealed that transplanted mutants swam greater distances over a 20-min period at 8 dpf compared to nontransplanted controls, which themselves were hypoactive relative to WT (Fig. 5 *A* and *B*). This rescue was mirrored at 4 wpf, with transplanted, microglia-depleted *rnaset2* mutants showing greater motor activity over a 10-min period of free-swimming, compared with sham controls (Fig. 5*C*). Notably, at this timepoint, nondepleted transplanted *rnaset2* animals were not assayed due to a failure of persistent engraftment in these animals by 4 wpf (Fig. 3 *E* and *F*). Interestingly, in microglia-depleted transplanted hosts, the extent of motor recovery was similar between juveniles which had persistent cell engraftment and those which did not—suggesting that there may be some residual benefits of transplantation shortly after these cells disappear from the brain (Fig. 5*D*). Strikingly, this behavioral improvement appeared to be accompanied by an increase in survival of transplanted *rnaset2* mutants at 4 wpf relative to microglia-depleted sham controls (Fig. 5 *E* and *F*). As such, macrophage transplantation has therapeutic benefits beyond embryonic stages lasting into juvenile stages in *rnaset2* mutants.

## Discussion

In this study, we have demonstrated that the replacement of diseased microglia with healthy macrophages can rescue *rnaset2* pathology at a molecular, cellular, and behavioral levels. We have demonstrated that transplanted macrophages can reach embryonic brains, adopt a microglia-like phenotype, and maintain engraftment for 4 to 10 wk post-transplantation. Transplanted macrophages compete with host microglia for the filling of the microglial niche, even when host microglia are deficient in their ability to clear inflammatory debris in the brain. Nonetheless, the presence of transplanted cells was able to normalize the antiviral response, clear apoptotic debris, and rescue the behavioral phenotypes shown by *rnaset2* mutants. RNA sequencing confirmed that macrophage transplantation rescues antiviral responses in *rnaset2* mutants at 4 wpf juvenile stages. As such, this work supports the hypothesis that supplementation of healthy microglia improves aspects of pathology in RNASET2-deficient leukodystrophy and that therapeutic targeting of the microglia may represent a future treatment avenue.

We have previously demonstrated that microglia-specific rescue of *rnaset2* expression can restore aspects of microglial function in *rnaset2* mutants—improving clearance of developmental apoptosis and restoring normal microglial morphology (26). The current study extends this finding—showing that the presence of healthy phagocytes in the brain can not only clear apoptotic debris but rescue the neuroinflammatory response and restore motor function through larval and juvenile stages. Indeed, multiple other studies have supported the finding that microglial replacement can improve aspects of pathology—including behavioral rescue—across various neurodegenerative diseases (44, 45). Additionally, treatment of neurological disorders with a cellular—rather than gene-based—therapy is particularly appealing in the context of microglial dysfunction, as many viral vehicles for gene delivery fail to efficiently target microglia at a whole-brain level (46, 47). Our study therefore identifies a cellular mechanism whereby deficient microglia drive pathology in *rnaset2* mutants and supports a cellular approach to targeting microglia dysfunction in RNASET2-deficient leukodystrophy, beyond the genetic strategies previously explored.

Our data also evidence the ability of whole kidney marrow–derived macrophages to infiltrate the brain, divide, and express microglia-specific markers. In the zebrafish, these cells are most similar to human bone marrow–derived macrophages and so may mimic peripheral blood cells following transplant. These cells have previously been demonstrated to infiltrate the brain following microglia depletion, during periods of inflammation or during embryogenesis (before the formation of the blood–brain barrier) (48). Due to the early timepoint at which transplants were performed in the *rnaset2* mutant embryos, it is likely that all three of these factors contributed to successful engraftment in our intervention. However, the true extent to which transplant-derived macrophages become microglia-like remains unclear. Previous studies have demonstrated that although macrophages derived from bone marrow and peripheral blood were able to engraft within the brain and express some microglia-specific markers, these transplant-derived cells remained transcriptionally distinct to host microglia even following robust engraftment (49, 50). As such, it seems that our results mirror the findings that transplant-derived macrophages can adapt to their new CNS environment and adopt key microglial phenotypes—but that they may remain distinct from their endogenous counterparts.

It is possible that the potential failure of HSC-derived macrophages to fully adopt a microglial identity contributes to their lack of longevity in the host brain. However, it is interesting to note that there are two distinct populations of microglia in the zebrafish throughout development—with embryonic microglia derived from yolk sac progenitors gradually replaced throughout juvenile stages by a distinct population of adult HSC-derived macrophages (37). This replacement is thought to be dependent on the arrival of the adult kidney marrow–derived second wave—with embryonic microglia persisting when HSC-derived microglia are genetically depleted (51). Similarly, we found that transplant-derived cells persisted for longer durations in hosts which lacked this HSC-derived microglial population (*csf1r^DM^*), suggesting that the arrival of endogenous adult macrophages into the brain may be the driver for transplanted cell clearance—despite these whole kidney marrow–derived cells being more similar in ontogeny to the HSC-derived adult microglia than yolk sac embryonic progenitors (31). As such, it is possible that our transplant-derived cells undergo such extensive reprogramming following engraftment that they are recognized as self, follow the same trajectory as host embryonic microglia and are cleared as the infiltrating adult microglia arrive.

It is interesting to note that, in order to achieve robust engraftment in *rnaset2* mutants, transient depletion of the embryonic microglial niche was required. We had initially speculated that the failure of *rnaset2*-mutant microglia to clear developmental apoptosis (among the first triggers to attract circulating monocytes to the CNS) would be sufficient for the recruitment of transplanted macrophages to the brain (43). However, without depletion of the embryonic microglial niche, the number of transplant-derived cells engrafted within host brains, and the expression of microglia markers by these cells, was much poorer than those with endogenous microglia depletion. This may have clinical implications, suggesting that myeloablation might be required in the context of transplantation in patients. However, despite these differences, the extent of pathological rescue was comparable between depleted and nondepleted hosts during larval stages, in terms of clearance of apoptotic debris, normalization of the antiviral response and behavioral recovery. As such, it seems the presence of any additional healthy phagocytes in the brain can have benefits in *rnaset2* larvae, regardless of microglial identity. Additionally, even in microglia-depleted hosts, the mean number of transplant-derived cells remained much lower than the number of microglia which might be expected in a healthy age-matched embryo—suggesting that transplant-derived cells may be particularly potent in their ability to clear neuropathology. Previous work has demonstrated that circulation-derived myeloid cells have an enhanced phagocytic ability relative to endogenous microglia (33). Therefore, it seems that transplanted macrophages may be well-suited to clearing apoptotic debris and ameliorating the neuroinflammatory response, regardless of their expression of microglial markers in *rnaset2* hosts.

Microglia replacement was sufficient to rescue the pathological antiviral and behavioral phenotypes of *rnaset2* mutants—however, future work is needed to assess the impact of this intervention on myelin structure and function. By 8 dpf (among the earliest timepoints used in this study), zebrafish show robust myelination which has been demonstrated to impact some behavioral functions (52, 53). As such, our finding that microglia replacement rescues *rnaset2* locomotion presents two possible explanations. The first is that transplantation supports myelin integrity in *rnaset2* mutants. Such a hypothesis could be explored in future studies using myelin- and oligodendrocyte transgenic reporter lines, electron microscopy, and MRI to investigate white matter function on a broader scale (53, 54). An alternative hypothesis is that *rnaset2* locomotion defects are independent of myelination state at the ages investigated. While myelination has been linked to time-sensitive escape responses following startle stimuli in the zebrafish, a multitude of other factors can impact free swimming behaviors, including neuroinflammation and viral infection (55, 56). Therefore, the interplay between transplant-derived cells, oligodendrocytes, and myelination remains unclear. Nonetheless, the rescue of locomotion in transplanted animals suggests that the microglia are an attractive target for the development of future interventions in RNASET2-deficient leukodystrophy.

In summary, our study highlights a cellular mechanism whereby microglia are the drivers of RNASET2-deficient neuropathology and suggests that microglia-directed approaches may have therapeutic benefits in leukodystrophy. We demonstrate that adult whole kidney marrow macrophages can engraft in host brains and undertake microglial phenotypes, both in WT animals and in our disease model. Restoring microglia function by macrophage transplantation was sufficient to rescue brain-wide autoimmune phenotypes and restore locomotor activity from embryonic to juvenile stages. In particular, this work suggests that cellular replacement strategies may have substantial impact in targeting the microglia, such as macrophage- or hematopoietic stem cell transplantation.

## Ethics Approval

All procedures involving zebrafish followed the Animal (Scientific Procedures) Act 1986 under the Home Office Project Licence (P254848FD and PP2981126).

## Supplementary Material

Appendix 01 (PDF)

Movie S1. Transplant derived cells can divide within microglia-depleted host brain. GFP-positive transplanted cells from the Tg(*fms*:GFP) reporter line within the brain of a 5dpf microglia-depleted zebrafish brain.

## Data Availability

RNA sequencing dataset have been deposited in SAR (PRJNA1047321), available at https://www.ncbi.nlm.nih.gov/bioproject/PRJNA1047321 (57). All study data are included in the article and/or supporting information.
