# Supplementary material for: Macrophage transplantation rescues RNASET2-deficient leukodystrophy by replacing deficient microglia in a zebrafish model
Source: Proc Natl Acad Sci U S A. 2024 May 16;121(21):e2321496121. doi: 10.1073/pnas.2321496121 (PMC11126979; doi:10.1073/pnas.2321496121)
Supplement: Supplementary file 1 — Appendix 01 (PDF) [file pnas.2321496121.sapp.pdf]

Supplementary Methods

Tissue clearing

Tissue clearing and immunohistochemistry methodology was adapted from Susaki *et al. Nature Protocols* 2015 (1) and Ferrero *et al. Cell Reports* 2018 (2).

For long-term assessment of transplant engraftment, successfully transplanted animals were raised according to standard zebrafish husbandry. Fish were checked weekly to observe any potential side effects of transplantation, and to maintain fish health. At the desired timepoint, fish were culled by decapitation with each head placed into 2ml 4% PFA and gently inverted to ensure the entire tissue was fully submerged, before leaving at 4°C overnight to ensure fixation of the tissue. Samples were then rinsed in autoclaved PBS and brains dissected in a Petri dish containing PBS placed on ice under a light microscope. Dissected brains were then gradually dehydrated using sequential 30-minute washes in methanol: 25% MeOH: 75% PBS; 50% MeOH: 50% PBS; 75% MeOH: 25% PBS; 100% MeOH). For long-term storage, samples were placed in fresh methanol and stored at –20°C to ensure preservation of tissue and quenching of fluorescent signal to minimise interference with subsequent staining.

CUBIC reagents were prepared according to Susaki *et al. Nature Protocols* 2015: CUBIC-1: 25% (by weight) urea (Sigma, 15604-1KG), 25% Quadrol (Sigma, 122262-1L), 15% Triton X-100 (Sigma, X100-500ML) in dH2O; CUBIC-2: 25% urea, 50% sucrose (Fisher, 10386100), 10% triethanolamine (Sigma, 90278-100ML) in dH2O (1).

At the time of clearing, samples were rehydrated from 100% methanol using serial MeOH dilutions in dH_2O, as PBS is known to interfere with clearing via CUBIC-1. At this stage, samples <14dpf were taken forward for immunohistochemistry without any additional clearing, due to their small size and transparency. For samples 28dpf and older, brains were then submerged in 1ml 50:50 CUBIC-1:dH_2O for up to six hours. Following full equilibration of the sample (confirmed by checking that the sample has sunk to bottom of the tube), 50:50 CUBIC-1:dH_2O was removed and 1ml CUBIC-1 was added. Samples were then left to incubate at room temperature for 3 days.

After CUBIC-1 clearance, samples were washed three times with PBS, before being immersed in 1ml 50:50 CUBIC-2:PBS and incubated for up to 24 hours. 50:50 CUBIC-2:PBS was then replaced with CUBIC-2, and samples left to incubate for 3 days for 28dpf animals, and 6 days for 42dpf and older.

After clearance, samples were ready for immunohistochemistry as described above. Samples were then digested in 1:500 proteinase K for 2 hours at room temperature, before three five-minute washes with PBS. Primary antibody incubation was 48 hours at 4°C. Secondary antibody incubation was 24 hours at 4°C. Finally, samples were incubated in 1:5000 DAPI solution (in PBS) for 60–90 minutes at room temperature, followed by thorough washes. Once staining was complete, the samples were re-cleared with CUBIC (overnight incubation with 50:50 CUBIC-2:PBS and 3–6 days in CUBIC-2) to ensure full transparency for imaging.

Samples were mounted in CUBIC-2 and aligned so that the dorsal surface of the brain was flush against the coverslip, before imaging with the Nikon W1 spinning disk confocal microscope. For each sample, a 500µm Z-stack was taken with 10x or 20x lens, using the Z-spacing recommended by NIS Elements (0.9µm for 20x images, 2.5µm for 10x images). Large images were taken using the tiling function (3x2 grid) within NIS elements. Samples from each timepoint were imaged on the same day to ensure fair comparison between groups. Analysis was performed with Arivis as described below.

Semi-automated quantification of cleared adult tissue

An automated pipeline was established using Arivis Vision 4D, which allows sophisticated segmentation of complex datasets. This quantification pipeline began by converting the ND2 file from the Nikon W1 Spinning Disk into an Arivis compatible SIS file. The optic tectum was manually identified as the region of interest from each whole brain image, as processing the whole image was beyond the capabilities of the analysis computers available in the Wolfson Light Microscope facility. The optic tectum was chosen as a region of interest to allow alignment with the embryonic data sets from other transplant assays (TUNEL etc.), and because this area remained robustly intact after dissection, immunohistochemistry and clearing while other more peripheral regions (such as the olfactory bulb or hindbrain and spinal cord) were frequently damaged. To begin quantification, a closing morphology filter was applied in other to smooth the appearance of otherwise highly ramified and irregular microglia to create a more uniform structure that can be better identified by subsequent segmentation tools. The 'blob finder' segmentation tool was then performed – an analysis tool suited to finding irregular, rounded objects. The parameters of this blob finder segmentation were adjusted to most accurately capture the relevant cell

population, using an average diameter of 30µm for objects in the GFP channel, and 15µm for 4C4-positive cells. Diameters were established by using manual measurement of cells in the raw image as a starting point, then adjusting the value until the segmentation most accurately mapped visible cells. The differing diameters set for each channel – despite labelling the same cell type – are attributed to the different cellular localisation of the marker proteins: *fms:GFP* being a cytoplasmic marker distributed throughout the cell, while the 4C4 antigen may be more discrete. Using these parameters, the blob finder segmentation was able to identify cells in three dimensions.

To remove any analysis artefacts, the resulting segments were filtered to exclude any objects with a volume larger than 5000μm^3 or less than 300μm^3. At the final stage, objects were manually filtered by intensity to account for differing staining efficiencies between timepoints to ensure the final object count best reflected the true number of cells.

To establish the co-localisation of 4C4 and GFP signal, a parent-child analysis was performed to quantify the number of GFP-positive objects that contained a 4C4-positive object, with a minimum overlap of 20%. Data was then exported into an Excel spreadsheet and visualised with GraphPad Prism.

<u>Bibliography</u>

1. E. A. Susaki, *et al.*, Advanced CUBIC protocols for whole-brain and whole-body clearing and imaging. *Nature Protocols* **10**, 1709–1727 (2015).
2. G. Ferrero, *et al.*, Embryonic Microglia Derive from Primitive Macrophages and Are Replaced by cmyb-Dependent Definitive Microglia in Zebrafish. *Cell Reports* **24**, 130–141 (2018).

Supplementary Video 1. Transplant-derived cells can divide within microglia-depleted host brains.

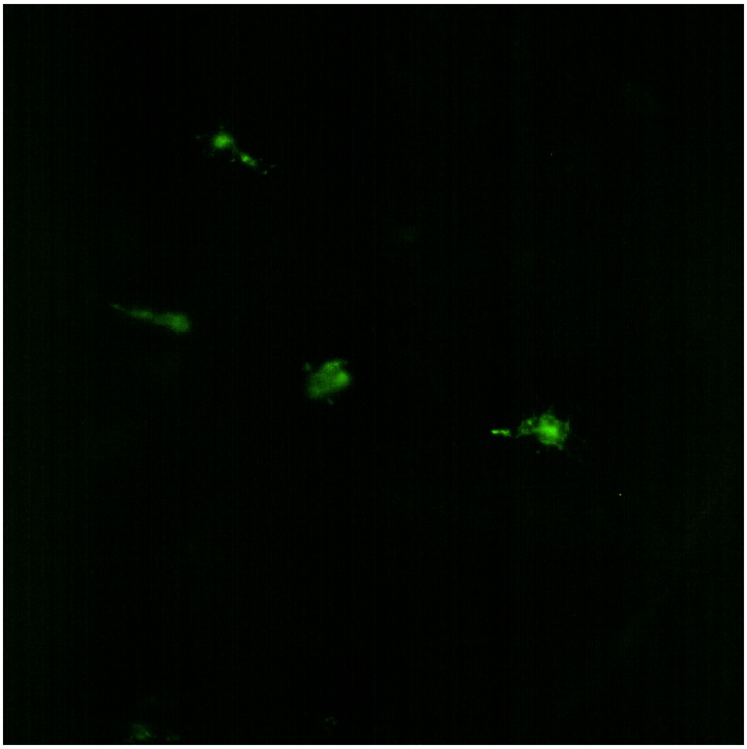

Supplemental video 1: Transplant derived cells can divide within microglia-depleted host brain. GFP-positive transplanted cells from the Tg(*fms*:GFP) reporter line within the brain of a 5dpf microglia-depleted zebrafish brain.

Supplementary Figure 1. Quantification of microglia depletion *irf8* crispants. a. Live screening of 5dpf *Tg(mpeg1:mCherry CAAX)sh378* embryos injected with double *irf8* targeting crRNAs revealed robust depletion of microglia compared with scrambled crRNA-injected controls. **b.** Representative images of *Tg(mpeg1:mCherry CAAX)sh378* embryos with normal microglia number (non-depleted), less than 15 microglia (incomplete depletion) and no microglia (microglia-depleted) in the optic tectum (dashed line). Scale bar represents 100µm. **c, d.** Immunohistochemistry against the microglia marker 4C4 revealed a significant reduction in microglia number in *irf8* crispants (microglia-depleted) *rnaset2* mutants relative to scrambled crRNA-injected (non depleted) controls at 5- **(c)** and 8dpf **(d).** Kruskal-Wallis test with Dunn's multiple comparisons. 3 biological repeats, n=14–39. **e, f.** Semi-automated quantification of the number of endogenous (4C4-positive, GFP-negative) microglia in the brains of *rnaset2* (4wpf) **(e)** and wild type **(f)** hosts with and without microglia depletion, visualised by tissue clearing and immunohistochemistry.

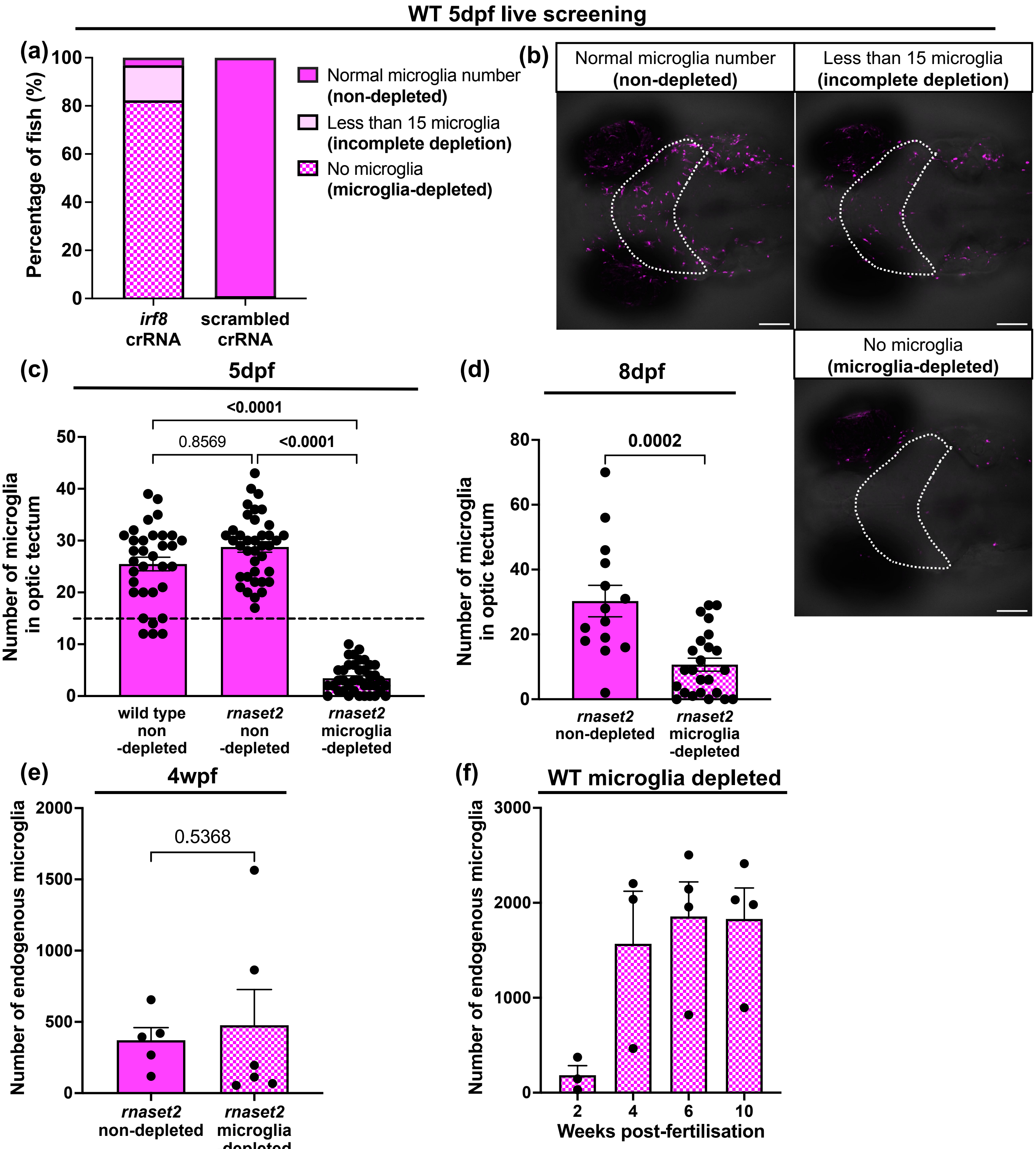

Supplementary Figure 2. Tissue clearing and immunohistochemistry of *fms:GFP* and *mpeg1:mCherry* brain. (a) GFP-positive cells in the optic tectum of *Tg(fms:GFP)sh377* animals (26wpf) **(b)** mCherry-positive cells in the optic tectum of *Tg(mpeg1:mCherryCAAX)sh378* animals (14wpf). Red dashed line indicates area imaged. Scale bar represents 200μm.

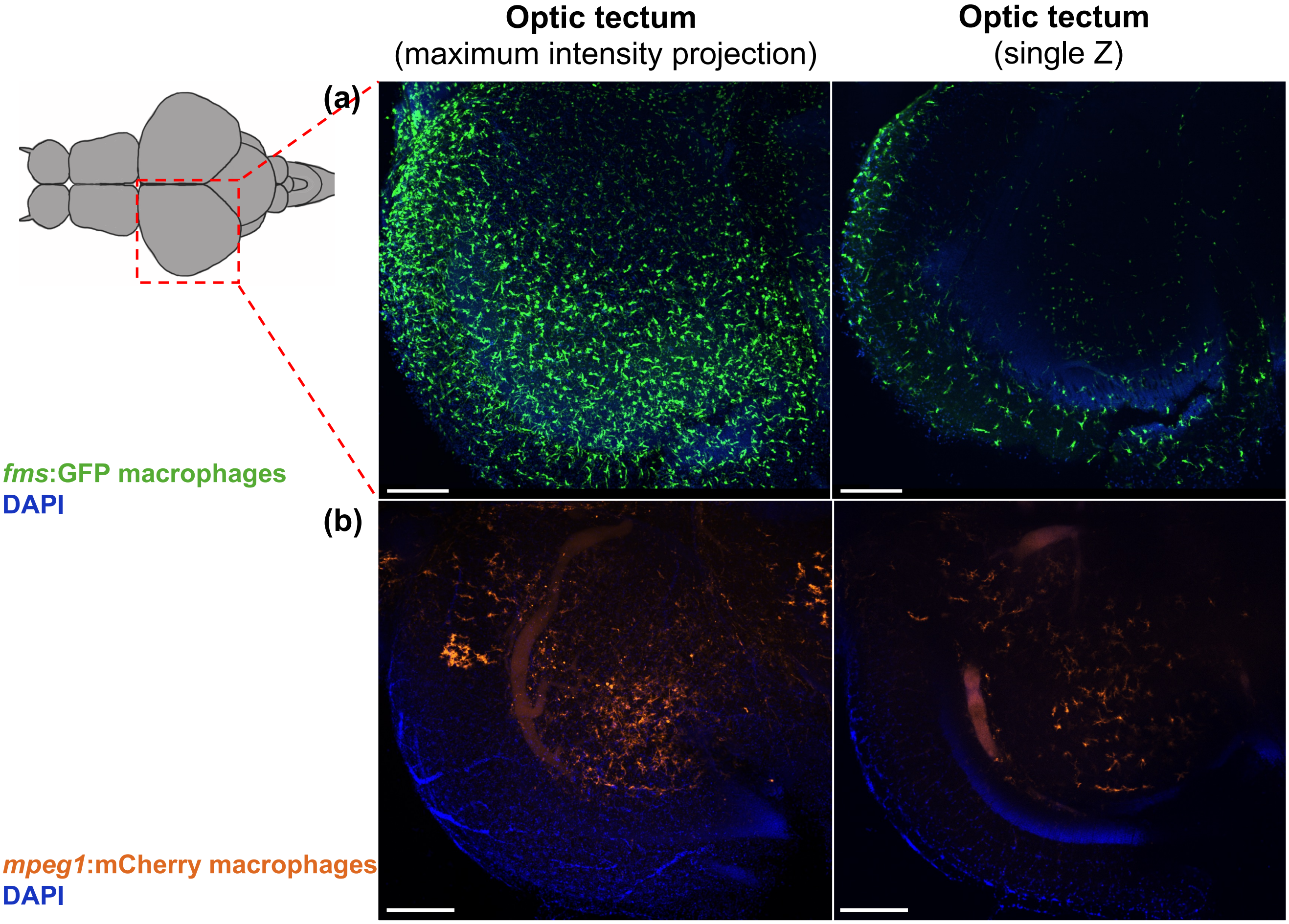

Supplementary Figure 3. Survival of wild type hosts following microglia depletion (a) and macrophage transplantation (b). Mann Whitney test. Three biological repeats.

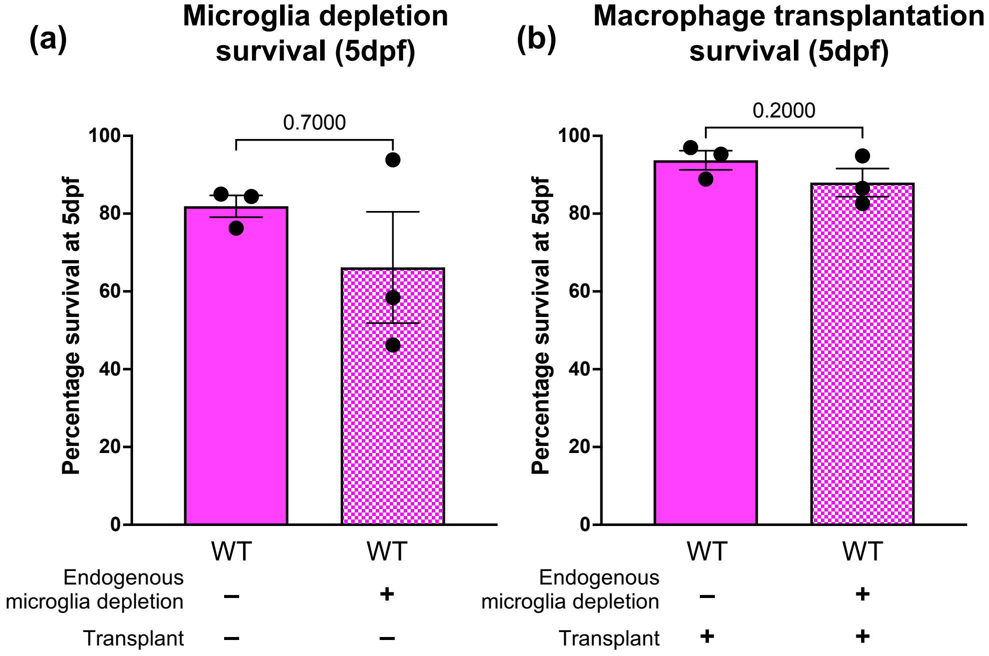

Supplementary Figure 4. Comparison of engraftment efficiency across multiple graft sources.

a, b. Percentage of fish with GFP-positive cells in the body **(a)** and brain **(b)** following systemic injection of *fms:GFP* cells from adult whole kidney marrow (*Tg(mpeg1:mCherry CAAX)sh378* hosts, 5dpf. 3 biological replicates, n=384–423). **c, d.** Percentage of fish with GFP-positive cells in the body **(c)** and brain **(d)** following systemic injection of *CD41:GFP* cells from adult whole kidney marrow (*Tg(mpeg1:mCherry CAAX)sh378* hosts, 7dpf. 1 biological replicate, n=37–43). **e, f.** Percentage of fish with GFP-positive cells in the body **(e)** and brain or CHT **(f)** following systemic injection of *fms:GFP* cells from whole embryo graft (*rnaset2*^{sh532} mutant and wild type sibling hosts, 7dpf. 1 biological replicates, n=34–72). dpf, days post-fertilization.

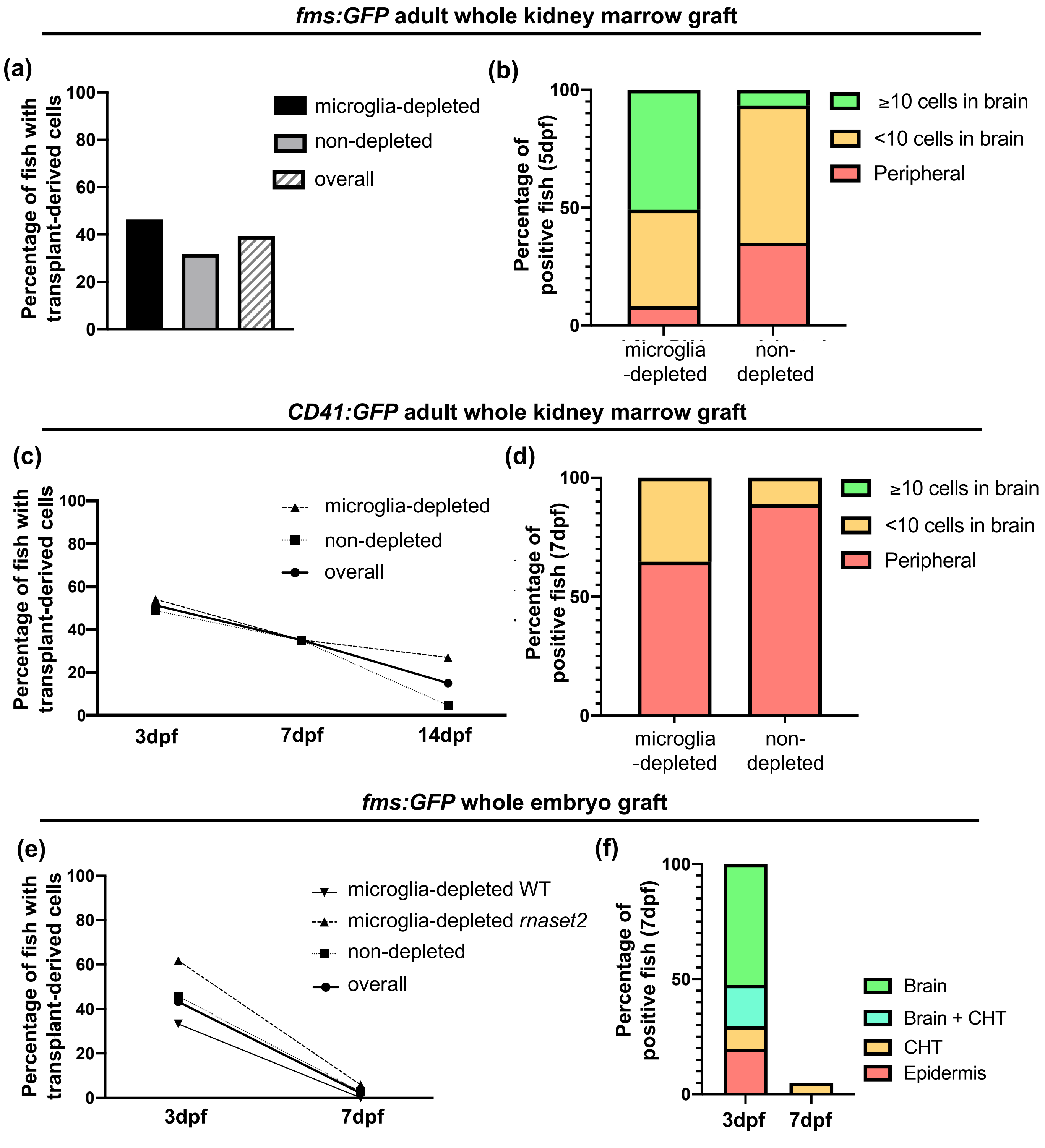

Supplementary Figure 5. Transplanted macrophages compete with endogenous cells to fill the microglia niche in *rnaset2* mutants. Quantification of the number of GFP-positive cells within *rnaset2* mutant and wild type host brains with and without microglia-depletion via targeting of *irf8*. Kruskal-Wallis test with Dunn's multiple comparisons, 3 biological replicates, n=60–90

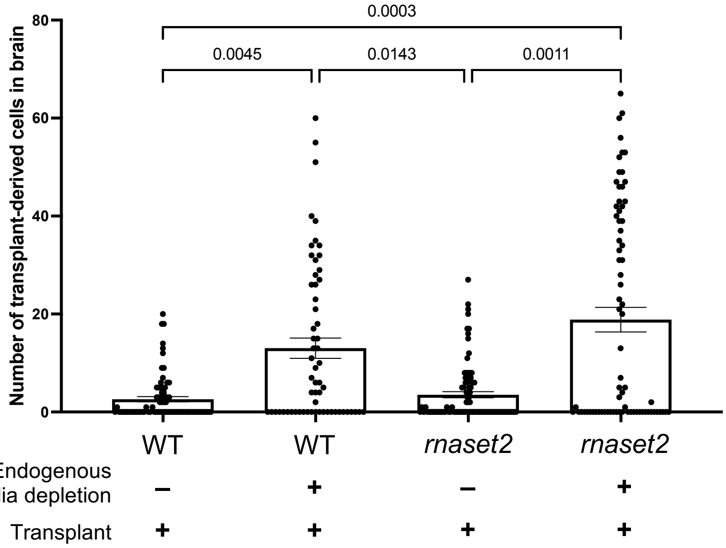

Supplementary Figure 6. Representative images of TUNEL counts for *rnaset2* transplanted hosts at 8dpf, with and without microglia depletion. Yellow dashed line indicates region of interest (optic tectum). Scale bar represents 100µm

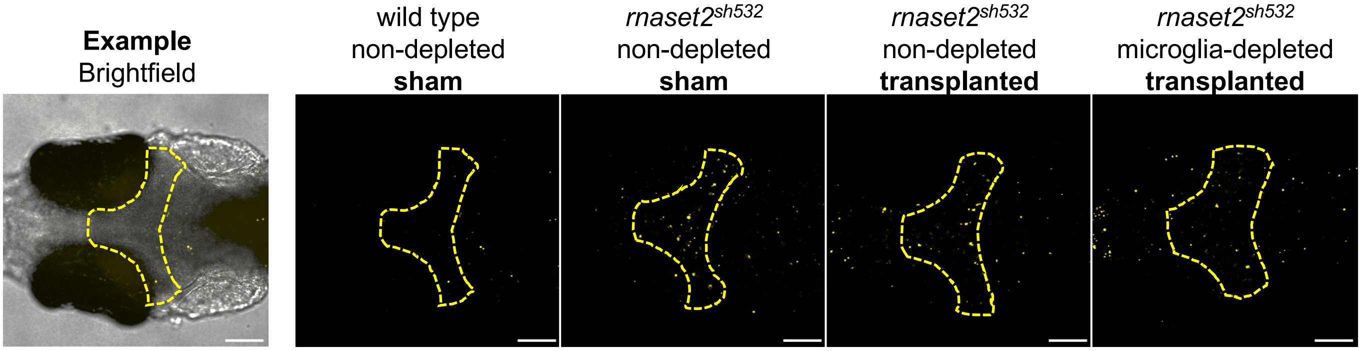

Supplementary Figure 7. Transplantation of healthy microglia does not increase cross-correction of rnaset2 in *rnaset2* mutant heads (a) nor trunks (b). Kruskal-Wallis test with Dunn's multiple comparisons. 3 biological replicates, 15 embryos pooled per replicate.

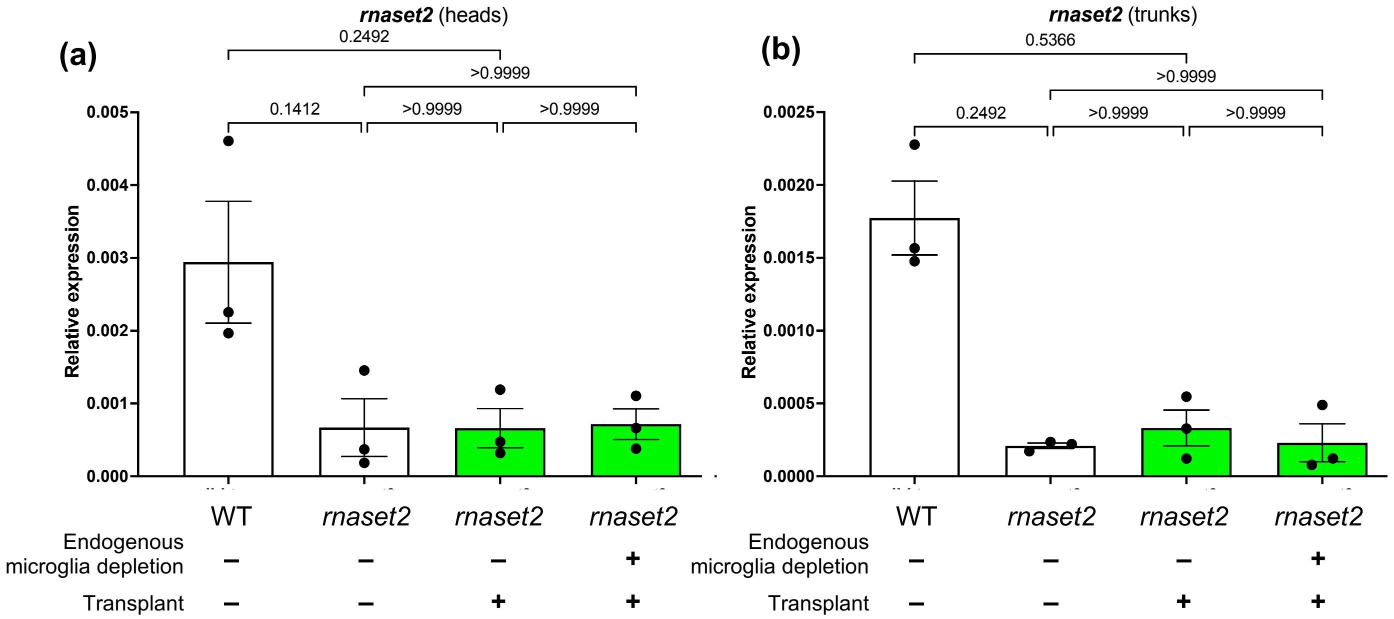

Supplementary Figure 8. Transplanted cells persist beyond 26wpf in *csf1r* double mutants. a. Tissue clearing and immunohistochemistry reveals that transplant-derived cells persist in microglia-deficient $csf1r^{DM}$ host brains until 26wpf. Scale bar represents 400µm. **b, c.** The percentage **(b)** and number **(c)** of microglia from transplant origin gradually decreases until transplanted cells are no longer detectable in $csf1ra^{-/-};csf1b^{+/+}$ and $csf1ra^{-/-};csf1b^{+/-}$ brains over 26 weeks, while transplanted cells remain engrafted at 26wpf in $csf1r^{DM}$.

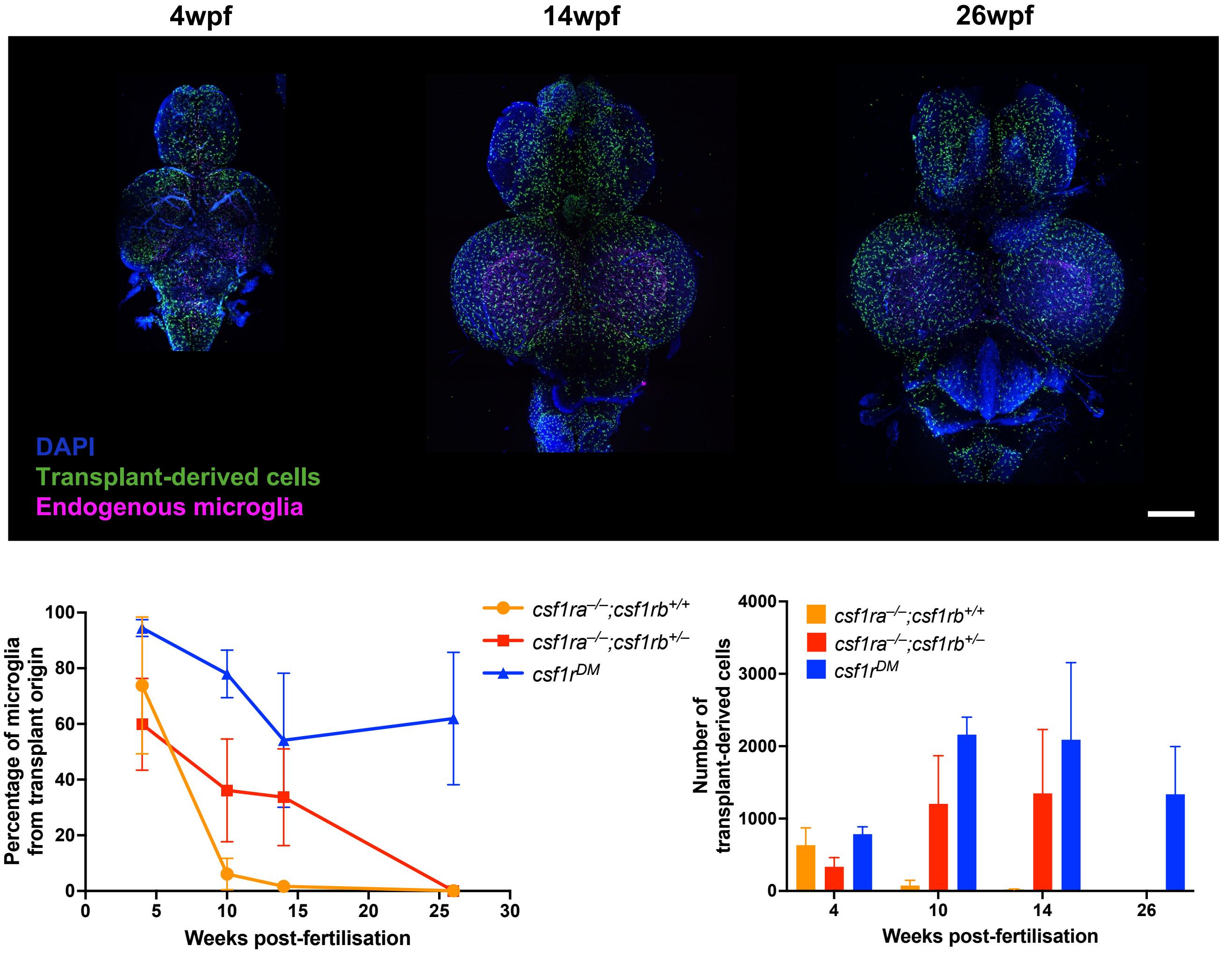

Supplementary Figure 9. RNA sequencing of microglia-depleted wild type sham, *rnaset2* sham and *rnaset2* transplanted brains.
a. Principle component analysis (PCA) plot reveals clustering of brains within each group. **b, c.** Complete heatmap of genes belonging to response to virus **(b)** and ISG15 antiviral mechanism **(c)** GO pathways for microglia-depleted animals. Fold change relative to wild type sham is indicated by color, with red indicating higher expression.

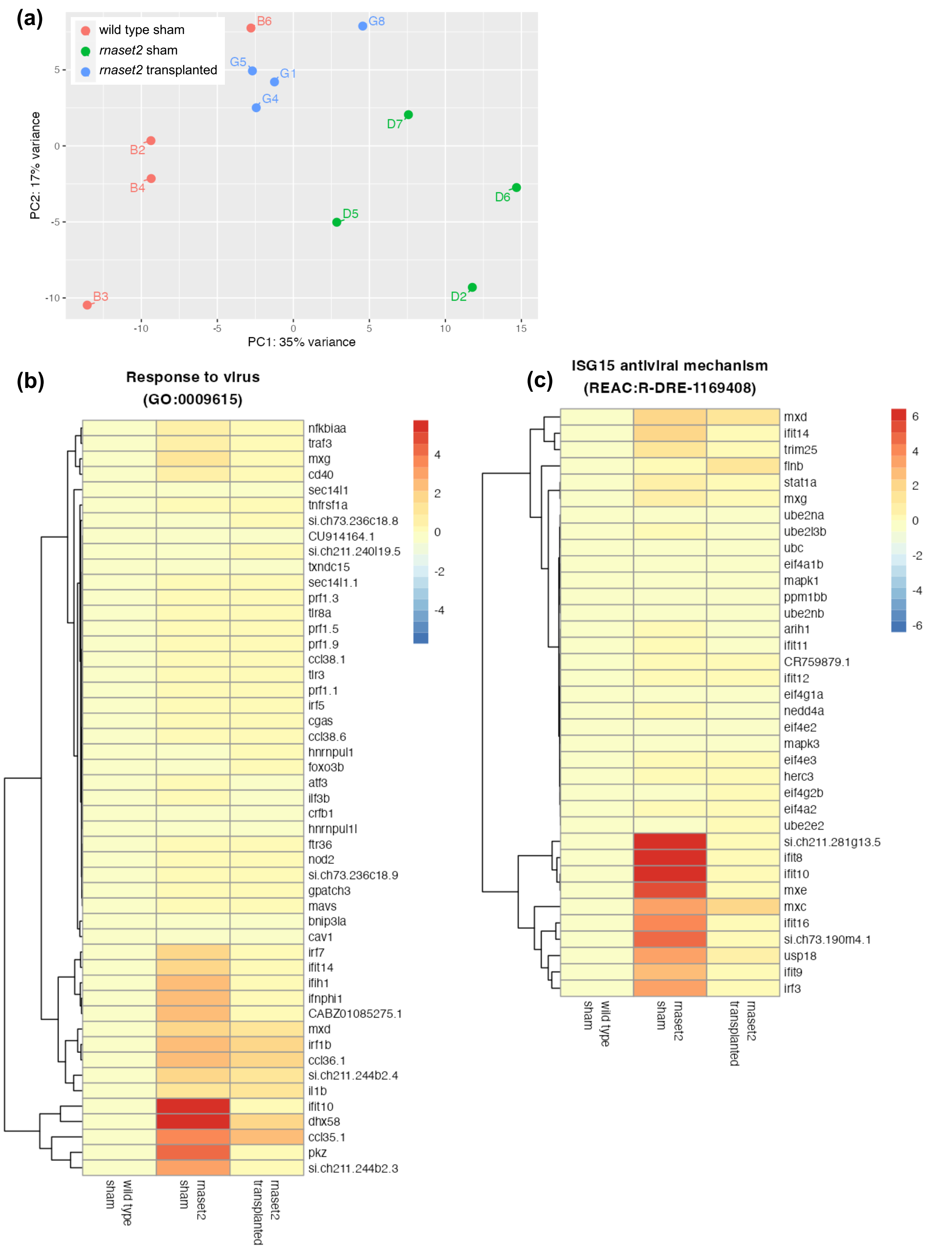

Supplementary Figure 10. RNA sequencing of non-microglia-depleted wild type sham, *rnaset2* sham and *rnaset2* transplanted brains.
a. Principle component analysis (PCA) plot reveals clustering of brains within each group. **b, c.** Volcano plot of differentially expressed genes between *rnaset2* sham versus wild type sham **(b)** and *rnaset2* transplanted versus *rnaset2* sham groups. Significantly differentially regulated genes are shown in red. The top 25 differentially expressed genes are annotated. **d.** Gene ontology (GO) plot showing significantly enriched pathways in *rnaset2* non-depleted transplanted animals compared with non-depleted sham controls, identified by gProfiler.

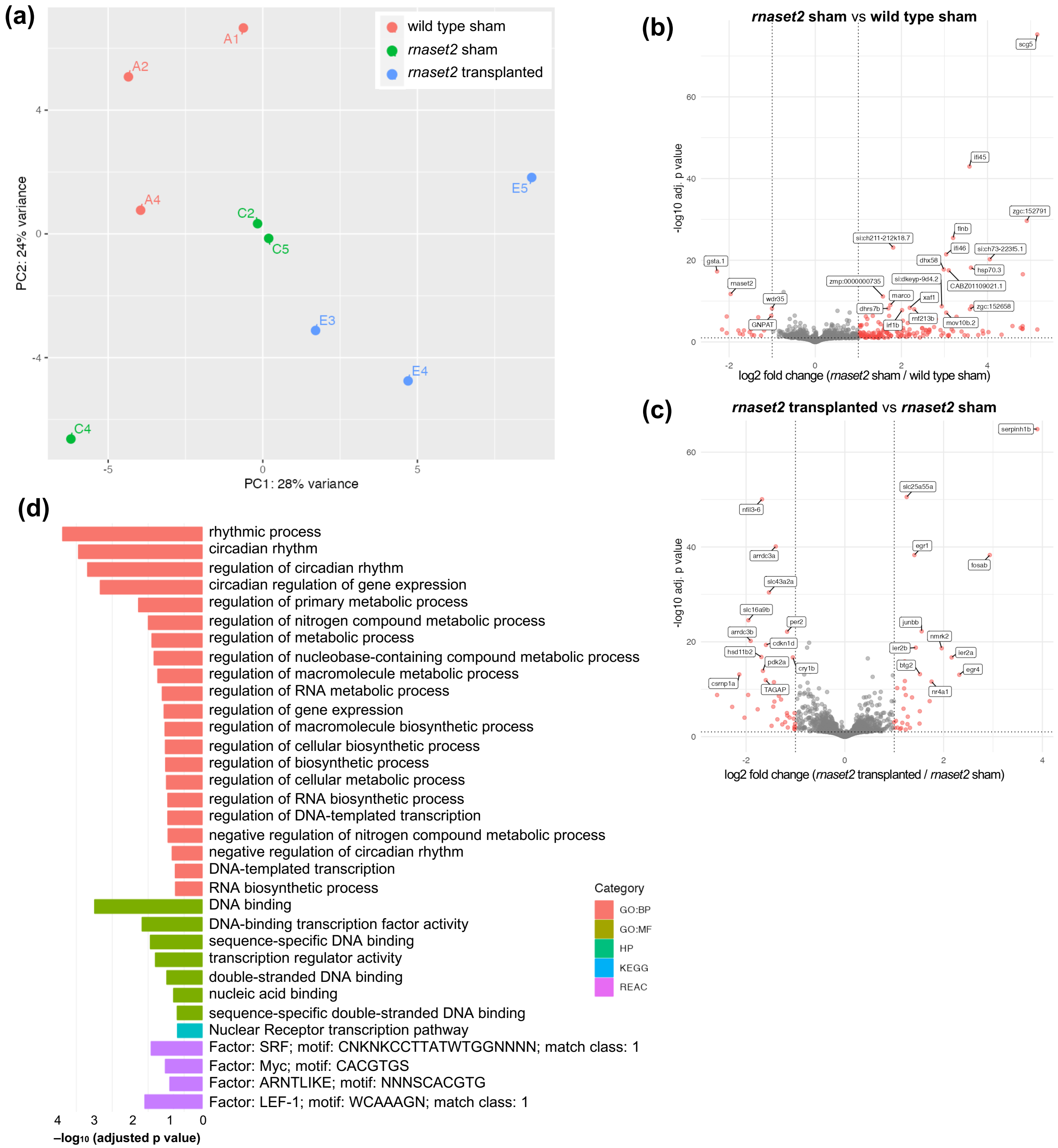

Supplementary Figure 11. Complete heatmap of genes belonging to response to virus and ISG15 antiviral mechanism GO pathways for nondepleted animals. Fold change relative to wild type sham is indicated by color, with red indicating higher expression.

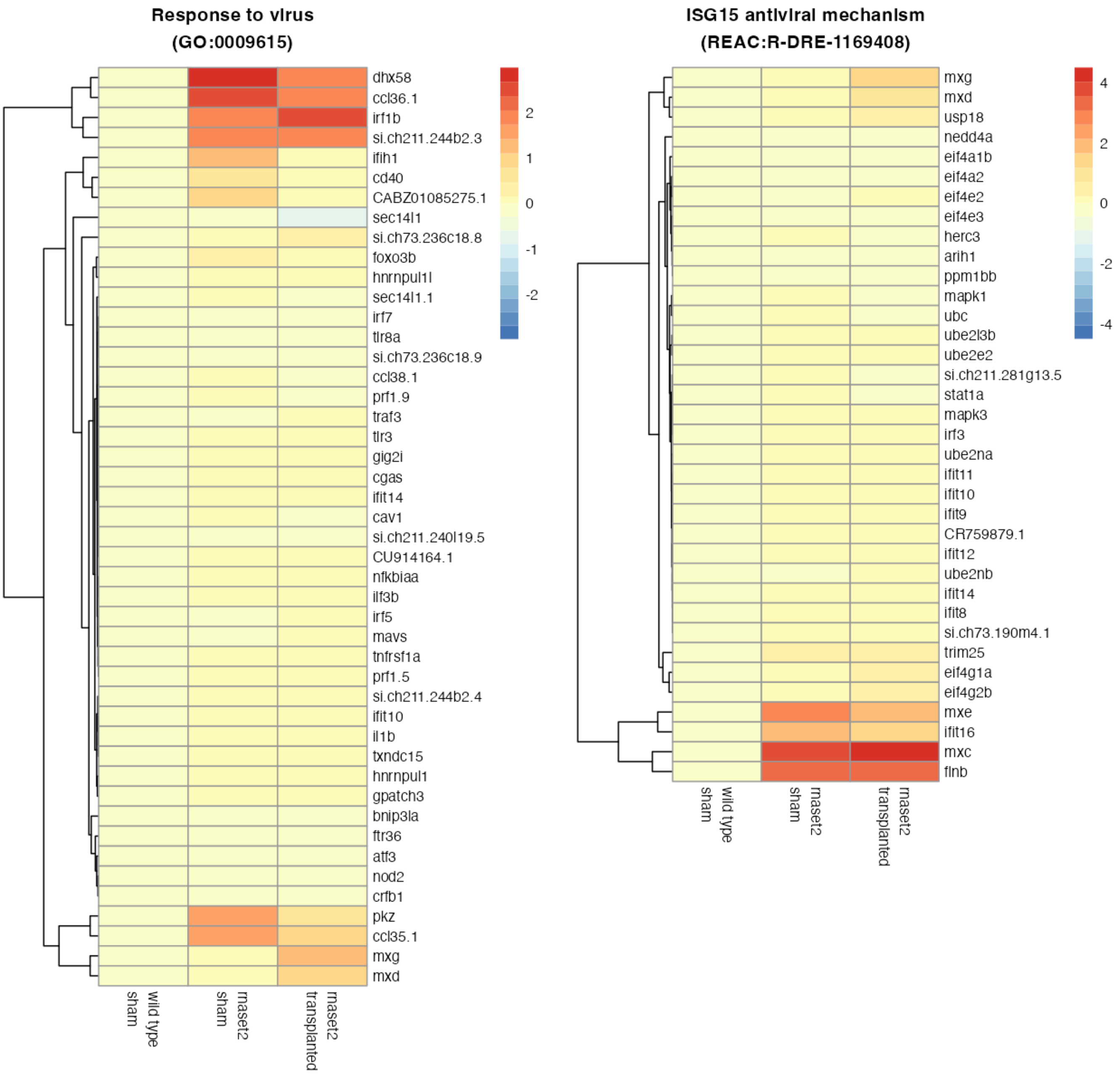

Table S1. CRISPR/Cas9 guide sequences

gene	guide name	sequence
irf8	irf8_crRNA_A	GCGGTCGCAGACTGAAACAGTGG
irf8	irf8_crRNA_B	GTCTACAAGATGAACTCGGG
n/a	scrambled_crRNA	GACCTGAGGGAGCAAGATCC

Table S2. qRT-PCR primers

gene	primer name	sequence
ef1α	ef1a_qPCR_fwd	CAGCTGATCGTTGGAGTCAA
ef1α	ef1a_qPCR_rev	TGTATGCGCTGACTTCCTTG
isg15	isg15_qPCR_fwd	AACTCGGTGACGATGCAGC
isg15	isg15_qPCR_rev	TGGGCACGTTGAAGTACTGA
mxa	mxa_qPCR_fwd	GACCGTCTCTGATGTGGTTA
mxa	mxa_qPCR_rev	GCATGCTTTAGACTCTGGCT
rnaset2	rnaset2_qPCR_fwd	TTACTCGCTGGAGGATGTG
rnaset2	rnaset2_qPCR_rev	GGTGTCTTCGCTGGACTTT
